# An Indirect Comparison of Diagnostic Accuracy for Seven Different SARS‐CoV‐2 Serological Assays: A Meta‐Analysis and Adjusted Indirect Comparison of Diagnostic Test Accuracy

**DOI:** 10.1111/irv.70155

**Published:** 2025-09-09

**Authors:** Minjie Zhang, Ying Zhao, Lijiang Fang, Weiwei Liang

**Affiliations:** ^1^ Department of Medical Laboratory Xian Yang Central Hospital Xianyang China; ^2^ Department of Medical Laboratory The Second Affiliated Hospital of Shaanxi University of Chinese Medicine Xianyang China; ^3^ Department of Medical Laboratory The Affiliated Hospital of Shaanxi University of Chinese Medicine Xianyang China

**Keywords:** adjusted indirect comparison, COVID‐19, meta‐analysis, SARS‐CoV‐2, serological assays

## Abstract

**Objectives:**

This study compared the diagnostic accuracy of seven different commercial serological assays for COVID‐19, using RT‐PCR as the gold standard, through meta‐analysis and indirect comparison.

**Methods:**

Fifty‐seven studies, published from November 2019 to June 2024, were included. The diagnostic performance of IgA, IgG, and total antibody assays for SARS‐CoV‐2 was assessed. The netmeta, rjags, and gemtc packages in R software were used for adjusted indirect comparison to calculate the relative diagnostic odds ratio (RDOR).

**Results:**

The pooled diagnostic odds ratio (DOR) for Abbott SARS‐CoV‐2 IgG was 542.81, for Elecsys Anti‐SARS‐CoV‐2 N was 1022.34, for Elecsys Anti‐SARS‐CoV‐2 total was 1701.56, for Euroimmun Anti‐SARS‐CoV‐2 IgA was 45.91, for Euroimmun Anti‐SARS‐CoV‐2 S1‐IgG was 190.45, for Euroimmun Anti‐SARS‐CoV‐2 N‐IgG was 82.63, and for LIAISON SARS‐CoV‐2 S1/S2 IgG was 178.73. The pooled DOR for IgG, IgA, and total antibody assays was 241.43, 45.91, and 1124.48. The pooled DOR for the antinucleocapsid antigen (anti‐N) was 604.29; for the antidomain of viral spike protein (anti‐S1) and the antirecombinant S1 and S2 (anti‐S1/S2) antigens, the pooled DORs were 119.88 and 178.73. ECLIA and CMIA methods had superior diagnostic performance compared with CLIA and ELISA, with no significant difference between ECLIA and CMIA. Total antibody assays showed the highest accuracy, followed by IgG, with IgA performing least effectively.

**Conclusions:**

Elecsys Anti‐SARS‐CoV‐2 total and N assays had the best overall diagnostic test accuracy. The diagnostic efficacy of the anti‐N total, IgG antibodies was statistically significantly higher than that of anti‐S IgG and IgA antibodies for COVID‐19.

## 1 Introduction

Severe acute respiratory coronavirus 2 (SARS‐CoV‐2) is the causative agent of coronavirus disease 2019 (COVID‐19). Real‐time reverse transcription polymerase chain reaction (RT‐PCR)‐remains the gold standard for COVID‐19 diagnosis [1]. However, its accuracy is influenced by factors such as viral replication timing and the quality of the sample [2]. Many suspected patients required repeated testing for several days before confirmation. Despite high analytical sensitivity, the RT‐PCR performance has been unsatisfactory in practice [3]. Particularly for asymptomatic infections and epidemiological studies. In contrast, serological testing is valuable for population surveillance, vaccine response monitoring, and as a supplement to RNA detection. Current evidence indicates that antibody tests can help confirm the present or past infection, although their clinical indications remain limited [4]. In addition, when combined with other laboratory and clinical findings, serological assays provide important insights for epidemiological monitoring and outbreak control. Measuring antibody response against SARS‐CoV‐2 has become a common strategy to assess exposure prevalence worldwide [5].

Serological testing is also useful for monitoring the spread of the SARS‐CoV‐2 pandemic in communities, identifying individuals who may serve as prophylactic or therapeutic neutralizing antibodies [6]. Several serological assays have been developed to detect SARS‐CoV‐2 antibodies in human serum or plasma, primarily targeting two major structural proteins, including the nucleocapsid (N) protein and the spike (S) glycoproteins. The S protein consists of S1 and S2 subunits, while the N protein is the most abundant viral protein, detectable early in blood and saliva during both asymptomatic and symptomatic infection. Moreover, the N protein is highly immunogenic and elicits strong antibody responses in COVID‐19 patients [7]. Burbelo et al. reported that N protein antibodies were more sensitive than the S glycoprotein antibody for detecting early infection [8]. Commercial immunoassays commonly measure single immunoglobulin classes (IgM, IgG or IgA) or total antibody levels. IgM antibodies typically appear within the first 2 weeks of symptom onset and decline soon after an infection clearance, indicating recent infection. IgG antibodies emerge later but persist for months to years [3]. Zervou et al. reported that IgA is the predominant immunoglobulin in early disease, with the highest level observed in patients with severe critical illness [9]. Recent findings suggest IgA is a promising early serological biomarker correlated with infection severity, and its role in controlling SARS‐CoV‐2 infection is becoming increasingly evident [10].

Serological testing for specific SARS‐CoV‐2 antibodies is a supplementary diagnostic tool and serves an essential role in evaluating immune responses to infection. Although practical, the reliability of serological assays depends on rigorous validation. In the early pandemic, numerous assays were rapidly developed and validated with limited samples, leading to suboptimal performance and low sensitivity. Many were unsuitable for clinical purposes or population monitoring. Larger manufacturers later introduced assays functional for mass testing, and while some comparative studies exist, large‐scale evaluations remain limited. This study aimed to assess the diagnostic accuracy of seven commercially available anti‐SARS‐CoV‐2 antibody assays to ensure result comparability and strengthen scientific evidence. Due to insufficient direct comparative data, we conducted an indirect comparison of their diagnostic performance. The assays comparison included the Abbott SARS‐CoV‐2 IgG assays from Abbott Laboratories (Abbott, Chicago, IL, USA), Elecsys Anti‐SARS‐CoV‐2 N (anti‐N) and Elecsys Anti‐SARS‐CoV‐2 total antibody assays from Roche Diagnostics (Roche, Basel, Switzerland), Euroimmun Anti‐SARS‐CoV‐2 IgA, Euroimmun Anti‐SARS‐CoV‐2 S1‐IgG, and Euroimmun Anti‐SARS‐CoV‐2 N‐IgG assays from EUROIMMUN (Lübeck, Germany), and the LIAISON SARS‐CoV‐2 S1/S2 IgG assay from DiaSorin (Saluggia, Italy). These assays are based on different detection methods, including electrochemiluminescence immunoassays (ECLIA), chemiluminescent microparticle immunoassays (CMIA), chemiluminescence immunoassays (CLIA), and enzyme‐linked immunosorbent assays (ELISA). Additionally, we aimed to evaluate the diagnostic accuracy of the target antibodies (total antibody, IgG, IgA), target antigens (N, S1, S1/S2), and assay methods through meta‐analysis and indirect comparison.

## 2 Materials and Methods

### 2.1 Search Strategy and Study Selection

Studies were retrieved from the Cochrane Library, PubMed, Embase, Web of Science, Chinese Biological Medicine Database (CBM), China National Knowledge Infrastructure (CNKI), and WANFANG databases. The search covered November 2019 to June 2024, using the strategy detailed in Appendix S1, and was limited to articles published in Chinese or English. Reference lists of included studies were also screened. This review followed the Preferred Reporting Items for Systematic Reviews and Meta‐Analyses (PRISMA) guidelines (Appendix S2).

### 2.2 Inclusion and Exclusion Criteria

The seven‐assay comparison included the Abbott SARS‐CoV‐2 IgG, Elecsys Anti‐SARS‐CoV‐2 N, Elecsys Anti‐SARS‐CoV‐2 total, Euroimmun Anti‐SARS‐CoV‐2 IgA, Euroimmun Anti‐SARS‐CoV‐2 S1‐IgG, Euroimmun Anti‐SARS‐CoV‐2 N‐IgG, and LIAISON SARS‐CoV‐2 S1/S2 IgG. All primary studies evaluating the performance of these assays were eligible. Inclusion criteria were as follows: (1) the studies using RT‐PCR as the diagnostic reference standard for SARS‐CoV‐2; (2) availability of patient's serum samples for serologic testing, with COVID‐19 diagnosis confirmed by RT‐PCR and/or imaging and laboratory findings; (3) negative control serum samples collected before 2019 or from individuals with PCR‐confirmed non‐COVID‐19 infection within the previous 6 months; (4) availability of true positive (TP), true negative (TN), false positive (FP), and false negative (FN) data, or sufficient data to derive these values; (5) only English and Chinese language manuscripts were assessed.

Exclusion criteria were as follows: (1) the studies assessing “in‐house” assay like enzyme immunoassay (EIA) or ELISA; (2) the studies on commercial assays for detecting SARS‐CoV‐2 neutralizing antibodies; (3) the studies focused on antibodies generated by vaccines; (4) the studies diagnosing COVID‐19 solely by clinical presentation or local guidelines without RT‐PCR confirmation; (5) studies lacking sufficient information on manufacturer/platform, assays method, immunoglobulin classes (IgG, IgA, or total antibody) or the SARS‐CoV‐2 targeting antigen used; (6) purely observational studies inappropriate for diagnostic accuracy testing; (7) studies with fewer than 30 negative control samples or reporting only analytical sensitivity; (8) studies from which data could not be extracted.

### 2.3 Data Extraction and Quality Assessment

Two reviewers independently extracted and assessed study quality using the Quality Assessment of Diagnostic Accuracy Studies (QUADAS‐2) tool, focusing on patient selection, index test performance, reference test performance, and flow/timing (for risk of bias). Extracted data included the first author, publication year, assay method, manufacturer/platform, target antigen, antibody type, COVID‐19 patient samples, and the samples of the control group. Quality assessment was conducted for each test method and study population.

### 2.4 Statistical Analysis

The meta‐analyses were performed by assay type, serological method, immunoglobulin class, and SARS‐CoV‐2 target antigen. Pooled sensitivity, specificity, positive likelihood ratio (PLR), negative likelihood ratio (NLR), diagnostic odds ratio (DOR), and the summary receiver operating characteristic (SROC) curves with 95% confidence intervals (CIs) were measured. The SROC curve based on 2 × 2 contingency tables illustrated sensitivity and specificity for each arm, while the area under the curve (AUC) was used to evaluate diagnostic accuracy. Indirect comparisons were performed against RT‐PCR as the reference standard, with the relative diagnostic odds ratio (RDOR) presented in a forest plot using R software (Parametric Technology Corporation). Publication bias was assessed with Deek's test.

## 3 Results

### 3.1 Study Characteristics

**FIGURE 1:**
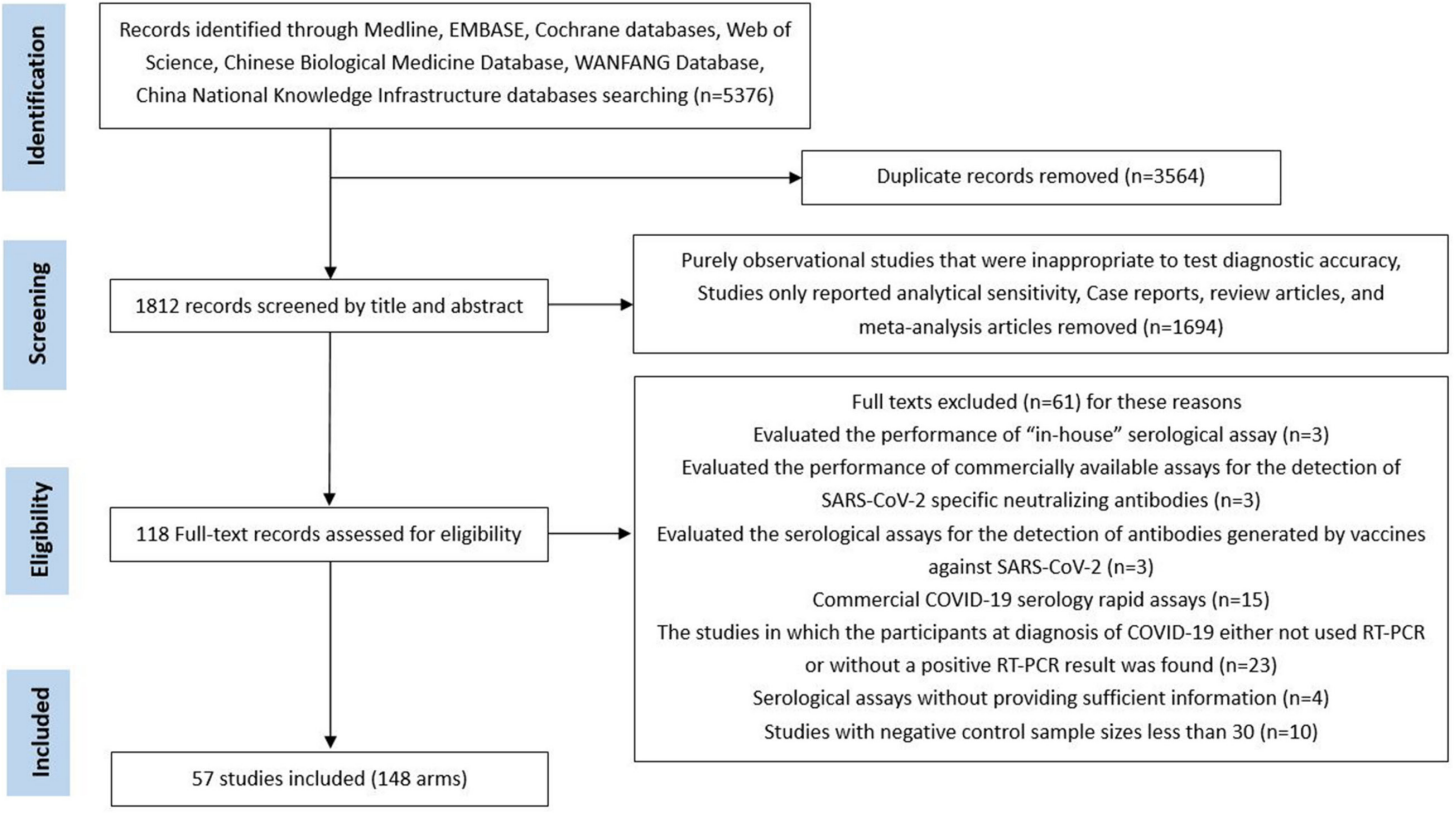
Flow diagram of selecting the literature and screening process.

A total of 5376 records were identified in the preliminary search. After removing 3564 duplicates and excluding 1694 based on title or abstract screening, 118 full‐text articles were assessed for eligibility. Finally, 57 articles [11, 12, 13, 14, 15, 16, 17, 18, 19, 20, 21, 22, 23, 24, 25, 26, 27, 28, 29, 30, 31, 32, 33, 34, 35, 36, 37, 38, 39, 40, 41, 42, 43, 44, 45, 46, 47, 48, 49, 50, 51, 52, 53, 54, 55, 56, 57, 58, 59, 60, 61, 62, 63, 64, 65, 66, 67], including 148 study arms, were included (Figure 1). These studies were published between 2020 and 2024. Detailed study characteristics are provided in Appendix S3: Table S1.

### 3.2 Quality Assessment of the Included Studies

**FIGURE 2:**
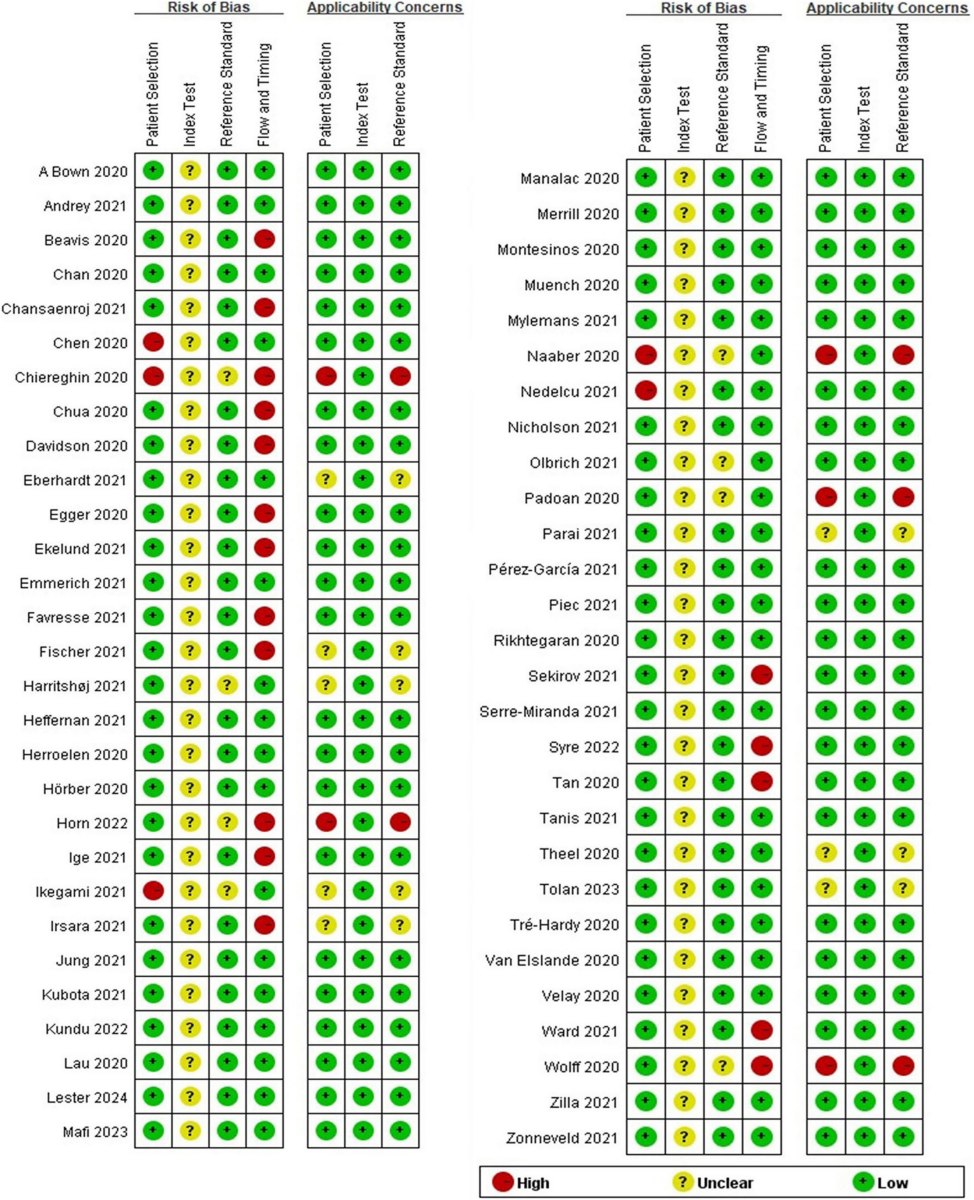
Risk of bias and applicability concerns summary.

Figure 2 presents the QUADAS‐2 assessments for the 57 included articles. Five articles were judged as “high risk” for bias in the patient selection, while eight articles were “unclear” in the reference standard domain, as COVID‐19 diagnosis was based solely on a positive RT‐PCR result regardless of symptoms, or samples were collected from recovered patients. Seventeen studies were rated “high risk” in the flow and timing domain. We defined an appropriate time interval of ≥ 7 days between the first positive RT‐PCR result and serum collection. Studies with shorter intervals were judged “high risk,” including Chansaenroj [15], Chiereghin [17], Davidson [19], Egger [21], Favresse [24], Fischer [25], Irsara [33], Sekirov [54], Tan [57], and Wolff [65]. Studies using different protocols or RT‐PCR reagents were also judged “high risk,” including Beavis [13], Chua [18], Ekelund [22], Horn [30], and Ward [64]. Ige [31] was rated “high risk” because four serum samples had insufficient volumes and were excluded. Syre [56] was similarly judged due to the inclusion of erum samples collected 1 year after PCR positivity. Full QUADAS‐2 assessments with explanations are shown in Appendix S4: Table S2.

### 3.3 Data Synthesis and Meta‐Analysis

**FIGURE 3:**
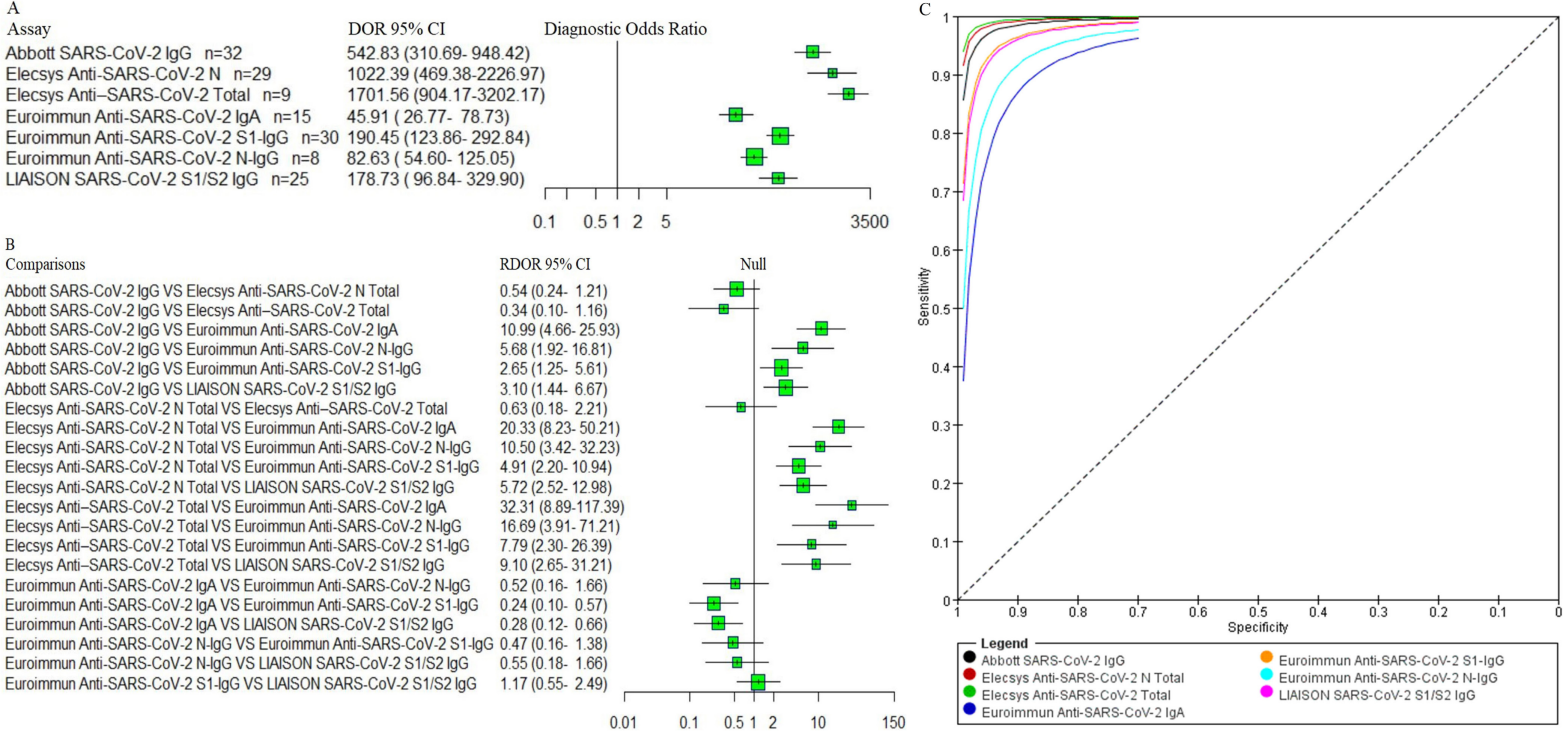
(A) The pooled DOR with 95% CIs of seven serological assays for diagnosing COVID‐19 were shown by forest plots. (B) Indirect comparison forest plots of RDOR with 95% CIs for all seven pairwise assays comparisons. (C) SROC plots of the seven serological assays. DOR, diagnostic odds ratio; CIs, confidence intervals; RDOR, relative diagnostic odds ratio; SROC, summary receiver operating characteristic curves.

Firstly, we evaluated the pooled performance metric (sensitivity, specificity, PLR, NLR, DOR, and AUC) of the seven serological assays (Table 1). SROC curves were constructed for all assays (Figure 3C) to visually assess overall diagnostic performance. The Euroimmun Anti‐SARS‐CoV‐2 IgA and Euroimmun Anti‐SARS‐CoV‐2 S1‐IgG assays showed pooled sensitivities of 80%, while Abbott SARS‐CoV‐2 IgG and LIAISON SARS‐CoV‐2 S1/S2 IgG both had 81%. The Euroimmun Anti‐SARS‐CoV‐2 N‐IgG assay had the lowest pooled sensitivity at 76%. In terms of specificity, Euroimmun Anti‐SARS‐CoV‐2 IgA performed poorly, while the other six assays exceeded 97%.

**TABLE 1:** The pooled analysis results of the reported seven serological assays.

Assay/subgroup	Pooled analysis results (95% CI)						Heterogeneity	
	Pooled sensitivity	Pooled specificity	Pooled PLR	Pooled NLR	AUC	DOR	*I* ^2^ (%)	*p*
Abbott SARS‐CoV‐2 IgG	0.81(0.75–0.85)	0.99(0.99–1.00)	158.20(88.00–284.50)	0.19(0.15–0.25)	0.99(0.97–0.99)	542.81(310.68–948.36)	77	< 0.01
Abbott SARS‐CoV‐2 IgG (CLIA)	0.75(0.64–0.84)	0.99(0.98–1.00)	137.80(40.30–470.90)	0.25(0.16–0.38)	0.97(0.96–0.98)	297.19(108.47–814.24)	82	< 0.01
Abbott SARS‐CoV‐2 IgG (CMIA)	0.83(0.77–0.87)	1.00(0.99–1.00)	169.50(92.60–310.20)	0.17(0.13–0.23)	0.99(0.98–1.00)	740.67(431.23–1272.15)	55	< 0.01
Abbott SARS‐CoV‐2 IgG (≥ 7 days)	0.80(0.75–0.85)	0.99(0.99–1.00)	151.60(83.90–273.70)	0.20(0.15–0.26)	0.99(0.97–0.99)	553.56(297.42–1030.31)	79	< 0.01
Abbott SARS‐CoV‐2 IgG (< 7 days)	0.82(0.64–0.93)	1.00(0.95–1.00)	275.60(15.30–4974.90)	0.18(0.08–0.39)	0.98(0.97–0.99)	502.04(111.76–2255.15)	69	0.01
Elecsys Anti‐SARS‐CoV‐2 N	0.85(0.79–0.90)	1.00(1.00–1.00)	617.90(180.50–2115.50)	0.15(0.10–0.21)	0.99(0.98–1.00)	1022.34(469.36–2226.83)	85	< 0.01
Elecsys Anti‐SARS‐CoV‐2 N (≥ 7 days)	0.87(0.81–0.91)	1.00(0.99–1.00)	417.70(135.30–1289.40)	0.13(0.09–0.20)	0.99(0.98–1.00)	1069.08(448.18–2550.18)	87	< 0.01
Elecsys Anti‐SARS‐CoV‐2 N (< 7 days)	0.69(0.65–0.73)	1.00(0.99–1.00)	191.98(54.45–676.91)	0.33(0.29–0.36)	0.99(0.98–1.00)	798.97(226.56–2817.55)^a^	0	0.71
Elecsys Anti‐SARS‐CoV‐2 total	0.89(0.83–0.93)	1.00(0.99–1.00)	303.50(156.30–589.30)	0.11(0.07–0.17)	1.00(0.99–1.00)	1701.56(904.15–3202.40)^a^	0	0.83
Elecsys Anti‐SARS‐CoV‐2 total (≥ 7 days)	0.88(0.80–0.93)	1.00(0.99–1.00)	294.00(143.50–602.40)	0.12(0.07–0.21)	1.00(0.99–1.00)	1533.36(774.76–3034.72)^a^	0	0.77
Elecsys Anti‐SARS‐CoV‐2 total (< 7 days)^c^	NA	NA	NA	NA	NA	NA	NA	NA
Euroimmun Anti‐SARS‐CoV‐2 IgA	0.80(0.76–0.84)	0.93(0.88–0.96)	10.90(6.60–17.70)	0.21(0.17–0.27)	0.91(0.89–0.94)	45.91(26.77–78.74)	83	< 0.01
Euroimmun Anti‐SARS‐CoV‐2 IgA (≥ 7 days)	0.80(0.74–0.86)	0.93(0.91–0.94)	11.00(9.20–13.00)	0.21(0.16–0.28)	0.94(0.91–0.96)	48.58(32.10–73.52)	59	< 0.01
Euroimmun Anti‐SARS‐CoV‐2 IgA (< 7 days)	0.81(0.77–0.84)	0.94(0.62–0.99)	13.50(1.60–114.00)	0.20(0.16–0.26)	0.81(0.78–0.85)	40.25(7.18–225.57)	93	< 0.01
Euroimmun Anti‐SARS‐CoV‐2 N‐IgG	0.76(0.70–0.81)	0.97(0.95–0.98)	22.10(15.30–31.90)	0.25(0.19–0.32)	0.97(0.95–0.98)	82.63(54.60–125.03)^a^	31	0.18
Euroimmun Anti‐SARS‐CoV‐2 N‐IgG (≥ 7 days) (omitting Favresse J)^b^	0.74(0.68–0.78)	0.96(0.94–0.98)	21.00(13.50–32.70)	0.27(0.23–0.33)	0.95(0.92–0.96)	69.34(44.48–108.09)^a^	0	0.47
Euroimmun Anti‐SARS‐CoV‐2 S1‐IgG	0.80(0.74–0.84)	0.98(0.97–0.99)	46.20(31.80–67.10)	0.21(0.16–0.27)	0.98(0.96–0.99)	190.45(123.87–292.83)	67	< 0.01
Euroimmun Anti‐SARS‐CoV‐2 S1‐IgG (≥ 7 days)	0.80(0.74–0.85)	0.98(0.98–0.99)	50.00(34.10–73.50)	0.20(0.15–0.27)	0.98(0.97–0.99)	214.43(137.29–334.91)	62	< 0.01
Euroimmun Anti‐SARS‐CoV‐2 S1‐IgG (< 7 days)	0.77(0.68–0.83)	0.97(0.94–0.99)	26.10(12.00–56.70)	0.24(0.18–0.33)	0.95(0.93–0.97)	94.96(29.99–300.70)	74	0.01
LIAISON SARS‐CoV‐2 S1/S2 IgG	0.81(0.75–0.86)	0.98(0.97–0.99)	38.80(25.20–59.80)	0.20(0.15–0.26)	0.98(0.96–0.99)	178.73(96.83–329.90)	87	< 0.01
LIAISON SARS‐CoV‐2 S1/S2 IgG (≥ 7 days)	0.81(0.75–0.86)	0.98(0.97–0.99)	41.10(25.20–67.00)	0.19(0.14–0.26)	0.98(0.96–0.99)	191.85(97.04–379.31)	88	< 0.01
LIAISON SARS‐CoV‐2 S1/S2 IgG (< 7 days)	0.78(0.73–0.82)	0.97(0.95–0.99)	29.67(14.52–60.62)	0.23(0.19–0.28)	0.95(0.92–0.97)	122.42(56.82–263.75)^a^	0	0.61

Abbreviation: NA: not specified.

^a^ The pooled DOR was calculated with a fixed effects model.

^b^ There was only one study (Favresse J) in the subgroup of “within 7 days.”

^c^ There were only two studies available, unable to pool data.

**FIGURE 4:**
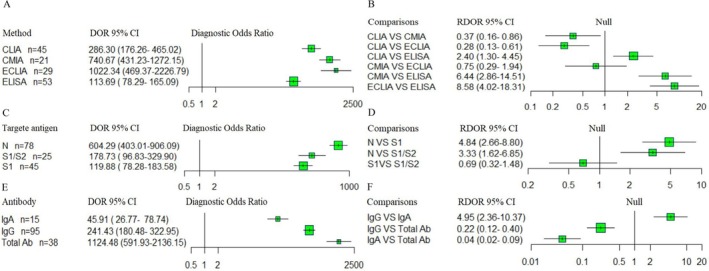
(A) The pooled DOR with 95% CIs of four methods for diagnosing COVID‐19 were shown by forest plots. (B) Indirect comparison forest plots of RDOR with 95% CIs for all four pairwise methods comparisons. (C) The pooled DOR with 95% CIs of antibody assays against the three targeting antigens were shown by forest plots. (D) Indirect comparison forest plots of RDOR with 95% CIs for all three pairwise targeting antigens comparisons. (E) The pooled DOR with 95% CIs of immunoglobulin were shown by forest plots. (F) Indirect comparison forest plots of RDOR with 95% CIs for all three pairwise immunoglobulin comparisons. DOR, diagnostic odds ratio; CIs, confidence intervals; RDOR, relative diagnostic odds ratio.

The Elecsys Anti‐SARS‐CoV‐2 total and Elecsys Anti‐SARS‐CoV‐2 N assays demonstrated the strongest diagnostic performance, with pooled PLR 303.50 and 617.90, pooled NLR of 0.11 and 0.15, and marked higher DORs (1701.56 and 1022.34 respectively) compared with other assays. The AUC values were 1.00 (95% CI: 0.99–1.00) for Elecsys Anti‐SARS‐CoV‐2 total, 0.99 (95% CI: 0.98–1.00) for Elecsys Anti‐SARS‐CoV‐2 N, 0.99 (95% CI: 0.97–0.99) for Abbott SARS‐CoV‐2 IgG, 0.98 (95% CI: 0.96–0.99) for Euroimmun Anti‐SARS‐CoV‐2 S1‐IgG, and LIAISON SARS‐CoV‐2 S1/S2 IgG. These findings indicate that the Elecsys Anti‐SARS‐CoV‐2 total, Elecsys Anti‐SARS‐CoV‐2 N, and Abbott SARS‐CoV‐2 IgG assays had the highest diagnostic accuracy. We further assessed pooled DOR (Figure 3A) and performed an indirect comparison of assay performance. A forest plot of RDOR value with 95% CIs was generated using R software (Figure 3B). No significant differences were observed among Elecsys Anti‐SARS‐CoV‐2 total, Elecsys Anti‐SARS‐CoV‐2 N, and Abbott SARS‐CoV‐2 IgG. Similarly, no significant differences were found in comparisons between Euroimmun Anti‐SARS‐CoV‐2 N‐IgG and either Euroimmun S1‐IgG (RDOR 0.47; 95% CI: 0.16–1.38) or LIAISON S1/S2 IgG (RDOR 0.55; 95% CI: 0.18–1.66), or between Euroimmun S1‐IgG and LIAISON S1/S2 IgG (RDOR 1.17; 95% CI: 0.55–2.49). However, diagnostic accuracy was significantly lower for Euroimmun IgA compared with Euroimmun S1‐IgG (RDOR 0.24; 95% CI: 0.10–0.57) and LIAISON S1/S2 IgG (RDOR 0.28; 95% CI: 0.12–0.66). We categorized the serological assays by the method: ECLIA, CMIA, ELISA, and CLIA. An indirect comparison was conducted to evaluate diagnostic accuracy across these techniques (Figure 4A). ECLIA demonstrated the highest diagnostic performance, with both ECLIA and CMIA outperforming CLIA and ELISA. No significant difference was observed between ECLIA and CMIA (RDOR = 0.75; 95% CI: 0.29–1.94). In contrast, RDOR values indicated a substantial difference in diagnostic accuracy among CMIA, CLIA, and ELISA (Figure 4B).

The pooled DOR of antibody assays against the SARS‐CoV‐2 antigen further supports these findings. Assays targeting the N antigen achieved the highest diagnostic accuracy (DOR = 604.29; 95% CI: 403.01–906.09) in a comparison of assays targeting the S1 antigen (DOR = 119.88; 95% CI: 78.28–183.58) and S1/S2 antigen (DOR = 178.73; 95% CI: 96.83–329.90) demonstrated lower but comparable accuracy, with no significant difference (RDOR = 0.69; 95% CI: 0.32–1.48) (Figure 4C,D).

We evaluated the diagnostic performance of assays targeting different antibody types (Figure 4E). The results showed that the pooled DOR for IgA was 45.91 (95% CI: 26.77–78.74), for IgG was 241.43 (95% CI: 180.48–322.95) and for total antibody was 1124.48 (95% CI: 591.93–2136.15) (Figure 4E). Moreover, indirect comparison results of RDOR with 95% CIs showed that total antibody had better diagnostic accuracy than IgG and IgA, with a significant difference. IgG showed significantly better diagnostic accuracy than IgA (Figure 4F).

### 3.4 Sensitivity Analysis

We examined heterogeneity across seven serological assays; significantly high heterogeneity was observed for Abbott SARS‐CoV‐2 IgG, Elecsys Anti‐SARS‐CoV‐2 N, Euroimmun Anti‐SARS‐CoV‐2 IgA, Euroimmun Anti‐SARS‐CoV‐2 S1‐IgG, and LIAISON SARS‐CoV‐2 S1/S2 IgG (Table 1). To explore potential sources, sensitivity analysis was conducted by sequentially omitting individual studies. Omitting a single study did not significantly affect the pooled DOR. However, heterogeneity decreased when “Kundu 2022 [36]” was omitted from Abbott SARS‐CoV‐2 IgG (*I* ^2^ = 58%, *p* < 0.01) (Appendix S5: Figure S1), and Elecsys Anti‐SARS‐CoV‐2 N (*I* ^2^ = 56%, *p* < 0.01) (Appendix S5: Figure S2), and when “Davidson 2020 [19]” was excluded from Euroimmun Anti‐SARS‐CoV‐2 IgA (*I* ^2^ = 63%, *p* < 0.01) (Appendix S5: Figure S3). Similarly, “Favresse 2021 [24]” accounted for heterogeneity in Euroimmun Anti‐SARS‐CoV‐2 N‐IgG (*I* ^2^ = 0%, *p* = 0.47 after omitting) (Appendix S5: Figure S4). The observed heterogeneity could be attributed to differences in assay methods, manufacturers, targeted antigens, and blood collection periods. Nevertheless, no significant heterogeneity was observed for Elecsys Anti‐SARS‐CoV‐2 total (*I* ^2^ = 0, *p* = 0.83) and Euroimmun Anti‐SARS‐CoV‐2 N‐IgG (*I* ^2^ = 31%, *p* = 0.18).

### 3.5 Subgroup Analysis

**FIGURE 5:**
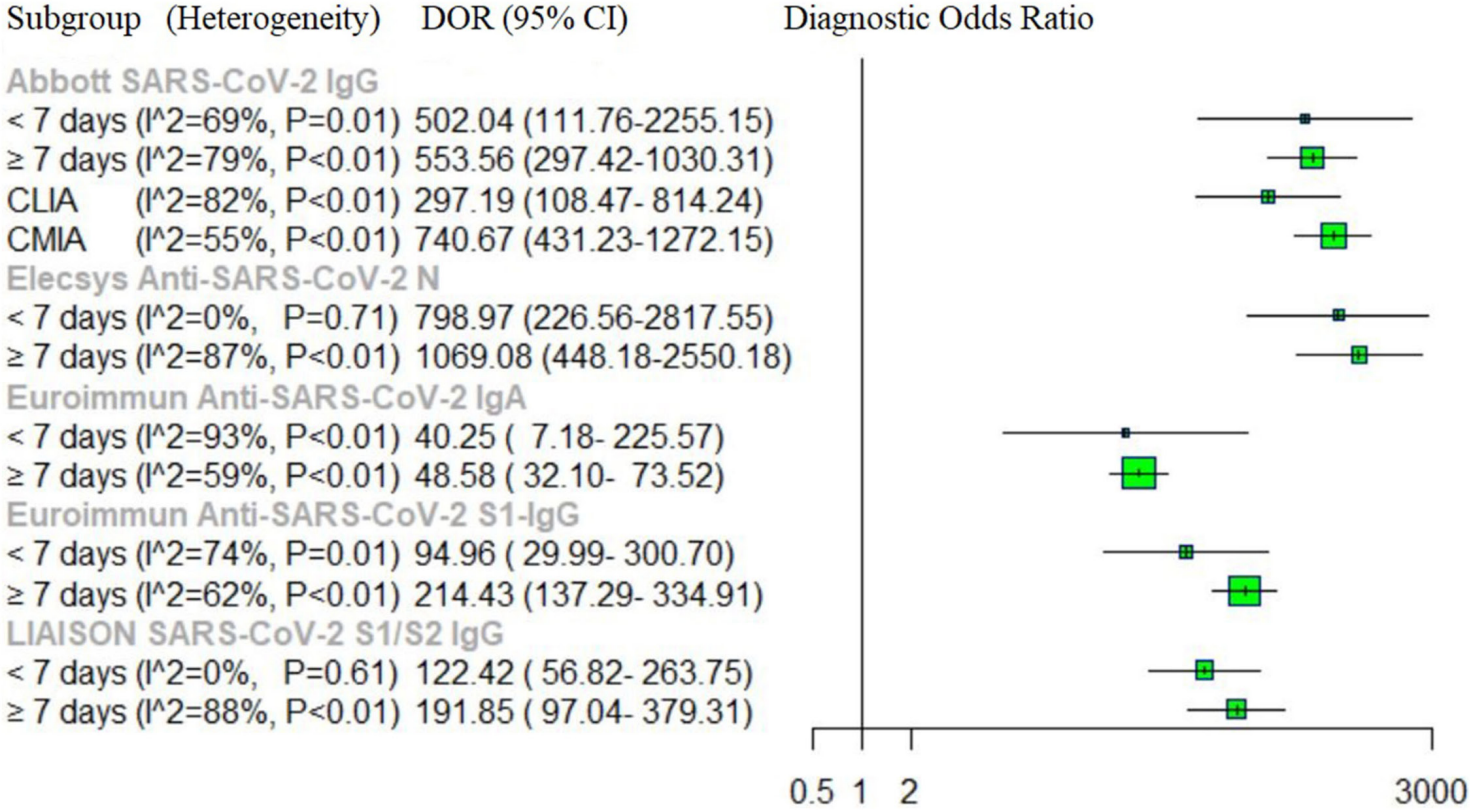
Subgroup analysis forest plot of DOR with 95% CIs for Abbott SARS‐CoV‐2 IgG. DOR, diagnostic odds ratio; CIs, confidence intervals. Harritshøj LH et al., serum samples were tested by two platforms: (A) Abbott Alinity; (B) Abbott Architect.

A subgroup analysis was conducted to evaluate the source of heterogeneity in Abbott SARS‐CoV‐2 IgG assays by automated method (divided into “CLIA group” and “CMIA group”). Results showed that higher heterogeneity in the CLIA group (*I* ^2^ = 82%, *p* < 0.01) compared with the CMIA group (*I* ^2^ = 55%, *p* < 0.01), suggesting variability linked to differences in automated instrumentation. Specifically, CMIA‐based Abbott Alinity platforms performed better than CLIA‐based Abbott Architect platforms. Additionally, a time interval subgroup analysis, based on duration between the first RT‐PCR positive result and serum collection (“≥ 7 days” vs. “within 7 days”) was performed for seven assays. The results indicated higher diagnostic efficiency in the“≥ 7 days” group for Abbott SARS‐CoV‐2 IgG, Elecsys Anti‐SARS‐CoV‐2 N, Euroimmun Anti‐SARS‐CoV‐2 IgA, Euroimmun Anti‐SARS‐CoV‐2 S1‐IgG, and LIAISON SARS‐CoV‐2 S1/S2 IgG (Figure 5).

### 3.6 Risk of Publication Bias Assessment

Publication bias was evaluated using Deek's test, and the corresponding funnel plot was constructed (Appendix S6). The slope coefficient result of the *t*‐value was −5.95 (*p* < 0.05), indicating significant publication bias.

## 4 Discussion

Determining seroprevalence is a critical component of the COVID‐19 response, and understanding the strengths and limitations of serological testing is essential for its application. Compared with molecular methods, serological assays generally offer faster turnaround times, higher throughput, and less workload. Moreover, assessing seroconversion allows the identification of individuals previously exposed to SARS‐CoV‐2, as well as those potentially protected, therefore supporting evidence‐based decisions on relaxing containment measures [68]. Several systematic reviews and meta‐analyses have evaluated the diagnostic characteristics of serological assays for SARS‐CoV‐2, focusing primarily on overall sensitivity and specificity [69, 70, 71], as well as study and patient‐level factors [72]. Building on this literature, our study provides pooled estimates of diagnostic accuracy for seven commercial assays, with RT‐PCR used as the reference standard. We further conducted an indirect comparison by calculating RDOR and visualized results using forest plots with 95% CI. Our study demonstrated that Elecsys Anti‐SARS‐CoV‐2 total and Elecsys Anti‐SARS‐CoV‐2 N assays achieved the highest diagnostic performance, considering both sensitivity and specificity, followed by the Abbott SARS‐CoV‐2 IgG assay. Importantly, no significant differences in diagnostic accuracy were observed among the Elecsys Anti‐SARS‐CoV‐2 total, Elecsys Anti‐SARS‐CoV‐2 N, and Abbott SARS‐CoV‐2 IgG assays. However, both the Euroimmun Anti‐SARS‐CoV‐2 S1‐IgG and LIAISON SARS‐CoV‐2 S1/S2 IgG assays showed significantly higher diagnostic accuracy compared with the Euroimmun Anti‐SARS‐CoV‐2 IgA assay. We also constructed an indirect comparison to assess the diagnostic accuracy of the four immunoassay techniques. In this analysis, CLIA (including Abbott SARS‐CoV‐2 IgG, Elecsys Anti‐SARS‐CoV‐2 total and LIAISON SARS‐CoV‐2 S1/S2 IgG assays), CMIA (including Abbott SARS‐CoV‐2 IgG assay), ECLIA (including Elecsys Anti‐SARS‐CoV‐2 N assay) and ELISA (Euroimmun Anti‐SARS‐CoV‐2 N‐IgG, Euroimmun Anti‐SARS‐CoV‐2 S1‐IgG, and Euroimmun Anti‐SARS‐CoV‐2 IgA assays) were evaluated. The DOR indicated that CLIA and ELISA demonstrated lower diagnostic performance compared with ECLIA and CMIA. Moreover, RDOR values confirmed that the diagnostic accuracy of CLIA and ELISA was statistically significantly lower than that of ECLIA and CMIA. The antibodies assessed in these assays primarily targeted the structural proteins of SARS‐CoV‐2. Among the four coronavirus structural proteins, the spike (S) and nucleocapsid (N) proteins have been identified as the most immunogenic and are widely used in the development of serological assays for COVID‐19 diagnosis [73]. The seven included assays differ both in the immunoglobulin classes detected (IgA, IgG, or total antibody) and the antigen employed for antibody recognition. Specifically, some assays such as Abbott SARS‐CoV‐2 IgG, Elecsys Anti‐SARS‐CoV‐2 N, Elecsys Anti‐SARS‐CoV‐2 total, and Euroimmun Anti‐SARS‐CoV‐2 N‐IgG were based on the N antigen. In contrast, others, including Euroimmun Anti‐SARS‐CoV‐2 IgA, Euroimmun Anti‐SARS‐CoV‐2 S1‐IgG, and LIAISON SARS‐CoV‐2 utilized the S1 or S1/S2 antigens. Assays detecting antibodies against the N antigen exhibited the highest diagnostic accuracy compared with those targeting S1 or S1/S2 antigens. The diagnostic accuracy of anti‐S1 and anti‐S1/S2 assays was comparable, with no statistically significant difference between the two.

SARS‐CoV‐2 infection generates antibodies to both the nucleocapsid (N) and spike (S) antigens, allowing detection through an assay targeting either antigen. However, vaccines are designed to elicit antibodies primarily against the S antigen or its receptor‐binding domain (RBD) [74], meaning that a positive anti‐S antibody result may reflect either prior infection or vaccination. Since few studies reported participants' vaccination status, it remains uncertain how vaccination influenced assay performance. Our analysis showed that ELISA assays (Euroimmun Anti‐SARS‐CoV‐2 S1‐IgG, Euroimmun Anti‐SARS‐CoV‐2 IgA) and CLIA assays (LIAISON SARS‐CoV‐2 S1/S2 IgG) using the S antigen demonstrated higher pooled sensitivity than ELISA assays targeting the N antigen (Euroimmun Anti‐SARS‐CoV‐2 N‐IgG). In contrast, the CMIA assays using the N antigen (Abbott Alinity SARS‐CoV‐2 IgG) outperformed S‐based ELISA and CLIA assays. Overall, the Elecsys Anti‐SARS‐CoV‐2 N assay and the Elecsys Anti‐SARS‐CoV‐2 total antibody assay, both targeting the N antigen and based on ECLIA and CLIA performance, exhibit the highest diagnostic accuracy. Although a general trend suggests slightly better performance for S antigen–based assays, results indicate that assay accuracy depends more on test design, methodology, timing of sample collection, and study population than on antigen choice alone. Due to limited reporting, the effect of vaccination on diagnostic accuracy could not be directly assessed.

To examine the effect of timing on diagnostic accuracy, studies were categorized into two groups: samples collected within 7 days and those ≥ 7 days after RT‐PCR positive results. Subgroup analysis showed that antibody assays had greater diagnostic efficacy when samples were obtained ≥ 7 days post‐infection, as reflected in a higher pooled DOR (Table 1). Notably, no heterogeneity was observed for Elecsys Anti‐SARS‐CoV‐2 N (*I* ^2^ = 0%, *p* = 0.71) and LIAISON SARS‐CoV‐2 S1/S2 IgG (*I* ^2^ = 0%, *p* = 0.61) within the ≥ 7 day subgroup, whereas heterogeneity remained high in the ≥ 7 day subgroup. Al Haddad et al. have reported that the sensitivity was low for four automated serological assays (Abbott, Euroimmun, Roche, and Snibe) within 7 days but increased with longer intervals [75]. Similarly, Zhao et al. found excellent sensitivity of the total antibody tests after 1 week, with rapid increases in seroconversion rates and antibody levels during the first 2 weeks [3]. These findings support the patterns observed in our subgroup analysis results.

Our study has several limitations. First, most previous evaluations of SARS‐CoV‐2 antibody assays reported that IgM‐based tests demonstrated the lowest diagnostic accuracy compared with other antibody isotypes. However, we could not assess IgM‐based assays due to the limited number of studies. Second, despite following a standardized search strategy, the absence of a universally accepted search term may have led to the omission of relevant articles. Third, indirect comparison provides weaker evidence than direct comparison; however, this approach was necessary given the scarcity of head‐to‐head studies.

## 5 Conclusions

This study compared seven anti‐SARS‐CoV‐2 antibody assays and identified three (Elecsys Anti‐SARS‐CoV‐2 total, Elecsys Anti‐SARS‐CoV‐2 N, and Abbott SARS‐CoV‐2 IgG) as demonstrating the highest diagnostic efficiency. Overall, anti‐N total, IgG antibodies showed statistically significantly higher diagnostic accuracy than anti‐S IgG and IgA antibodies. Furthermore, assays based on ECLIA, CMIA, and CLIA platforms exhibited higher accuracy than ELISA assays, suggesting they are a more reliable option for large‐scale testing, surveillance of virus spread, and monitoring herd immunity. Although vaccine‐induced antibodies may influence performance, our findings indicate that the choice of antibody target and detection method plays a more decisive role in diagnostic accuracy than the antigen type alone.

## Author Contributions

**Minjie Zhang:** conceptualization; data curation; formal analysis; investigation; methodology; writing – original draft. **Ying Zhao:** conceptualization; data curation; formal analysis. **Lijiang Fang:** conceptualization; data curation; formal analysis; writing – review and editing. **Weiwei Liang:** conceptualization; data curation; methodology; formal analysis.

## Conflicts of Interest

The authors declare no conflicts of interest.

## Peer Review

The peer review history for this article is available at https://www.webofscience.com/api/gateway/wos/peer‐review/10.1111/irv.70155.

## Supporting information


**Appendix S1:** Detailed search strategy. (DOC)

**Appendix S2:** PRISMA 2020 Checklist. (PDF)

**Appendix S3:** Table S1 Characteristics of the included studies. (DOC)

**Appendix S4:** Table S2 Quality assessment of QUADAS‐2 tool with explanation. (DOC)

**Appendix S5:** Sensitivity analysis for Abbott SARS‐CoV‐2 IgG, Elecsys Anti‐SARS‐CoV‐2 N, Euroimmun Anti‐SARS‐CoV‐2 IgA and Euroimmun Anti‐SARS‐CoV‐2 N‐IgG. (DOC)

**Appendix S6:** Funnel chart of Deek's test. (DOC)

## Data Availability

The data supporting this study's findings are available from the corresponding author, Lijiang Fang, upon reasonable request.
