# Supplementary material for: An Indirect Comparison of Diagnostic Accuracy for Seven Different SARS‐CoV‐2 Serological Assays: A Meta‐Analysis and Adjusted Indirect Comparison of Diagnostic Test Accuracy
Source: Influenza Other Respir Viruses. 2025 Sep 9;19(9):e70155. doi: 10.1111/irv.70155 (PMC12418076; doi:10.1111/irv.70155)
Supplement: Supplementary file 1 — Appendix S1: Detailed search strategy. (DOC) [file IRV-19-e70155-s005.doc]

**Appendix 1.** Detailed search strategy

| **China National Knowledge Infrastructure** |
| --- |
| (SU=新型冠状病毒肺炎OR SU=新冠肺炎OR SU=新型冠状病毒OR SU=COVID 19 OR SU=严重急性呼吸综合征冠状病毒2 OR SU=2019‐nCoV OR SU=SARS‐CoV‐2) AND (SU=Anti-SARS-CoV-2 OR SU=抗体 OR SU=血清学试剂 OR SU=血清抗体试剂 OR SU=SARS-CoV-2抗体试剂 OR SU=IgG试剂OR SU=IgA试剂 OR SU=总抗体 OR SU=抗SARS-CoV-2抗体 OR SU=Elecsys Anti-SARS-CoV-2 N OR SU=Elecsys Anti-SARS-CoV-2 Total OR SU=Elecsys Anti-SARS-CoV-2 总抗体 OR SU=Abbott SARS-CoV-2 IgG OR SU=Euroimmun Anti-SARS-CoV-2 S1-IgG OR SU=Euroimmun Anti-SARS-CoV-2 IgA OR SU=Euroimmun Anti-SARS-CoV-2 N-IgG OR SU=LIAISON SARS-CoV-2 S1/S2 IgG OR SU=电化学发光免疫分析 OR SU=化学发光微粒子免疫分析OR SU=化学发光免疫分析OR SU=酶联免疫吸附试验 OR SU=ECLIA OR SU=CMIA OR SU=CLIA OR SU=ELISA OR SU=SARS‐CoV‐2核衣壳蛋白OR SU=SARS‐CoV‐2刺突蛋白OR SU=SARS‐CoV‐2刺突蛋白S1亚基和刺突蛋白S2亚基OR SU=SARS‐CoV‐2重组刺突蛋白S1/S2亚基) |
| **Chinese Biological Medicine Database** |
| #1"新型冠状病毒肺炎"[Unweighted: extended] OR "新冠肺炎"[Unweighted: extended] OR "新型冠状病毒"[Unweighted: extended] OR "COVID 19"[Unweighted: extended] OR "严重急性呼吸综合征冠状病毒2"[Unweighted: extended] OR "2019‐nCoV"[Unweighted: extended] OR "SARS‐CoV‐2"[Unweighted: extended]  #2"Anti-SARS-CoV-2"[Common words: Intelligence] OR "抗体"[Common words: Intelligence] OR "血清学试剂"[Common words: Intelligence] OR "血清抗体试剂"[Common words: Intelligence] OR "SARS-CoV-2抗体试剂"[Common words: Intelligence] OR "IgG试剂"[Common words: Intelligence] OR "IgA试剂"[Common words: Intelligence] OR "总抗体"[Common words: Intelligence] OR "抗SARS-CoV-2抗体"[Common words: Intelligence]  #3#1 And #2  #4"Elecsys Anti-SARS-CoV-2 N "[Common words: Intelligence] OR "Elecsys Anti-SARS-CoV-2 Total"[Common words: Intelligence] OR "Elecsys Anti-SARS-CoV-2 总抗体"[Common words: Intelligence] OR "Abbott SARS-CoV-2 IgG"[Common words: Intelligence] OR "Euroimmun Anti-SARS-CoV-2 S1-IgG"[Common words: Intelligence] OR "Euroimmun Anti-SARS-CoV-2 IgA"[Common words: Intelligence] OR "Euroimmun Anti-SARS-CoV-2 N-IgG"[Common words: Intelligence] OR "LIAISON SARS-CoV-2 S1/S2 IgG"[Common words: Intelligence]  #5(("电化学发光免疫分析"[Unweighted: extended]) OR "化学发光微粒子免疫分析"[Unweighted: extended]) OR "化学发光免疫分析"[Unweighted: extended] OR "酶联免疫吸附试验"[Unweighted: extended] OR "ECLIA"[Unweighted: extended] OR "CMIA"[Unweighted: extended] OR "CLIA"[Unweighted: extended] OR "ELISA"[Unweighted: extended]  #6"SARS‐CoV‐2核衣壳蛋白"[Common words: Intelligence] OR "SARS‐CoV‐2刺突蛋白"[Common words: Intelligence] OR "SARS‐CoV‐2刺突蛋白S1亚基和刺突蛋白S2亚基"[Common words: Intelligence] OR "SARS‐CoV‐2重组刺突蛋白S1/S2亚基"[Common words: Intelligence]  #7#1 AND #4  #8#1 AND #5  #9#1 AND #5 AND #6 AND 2019-2024[date] |
| **Wanfang** |
| theme:("新型冠状病毒肺炎" or "新冠肺炎" or "新型冠状病毒" or “COVID 19” or “严重急性呼吸综合征冠状病毒2” or “2019‐nCoV” or “SARS‐CoV‐2”) and theme:("Anti-SARS-CoV-2" or "抗体" or "血清学试剂" or "血清抗体试剂" or " SARS-CoV-2抗体试剂" or " IgG试剂" or " IgA试剂" or "总抗体" or "抗SARS-CoV-2抗体" or "Elecsys Anti-SARS-CoV-2 N" or "Elecsys Anti-SARS-CoV-2 Total" or "Elecsys Anti-SARS-CoV-2 总抗体" or "Abbott SARS-CoV-2 IgG" or "Euroimmun Anti-SARS-CoV-2 S1-IgG" or "Euroimmun Anti-SARS-CoV-2 IgA" or "Euroimmun Anti-SARS-CoV-2 N-IgG" or "LIAISON SARS-CoV-2 S1/S2 IgG") and theme:("电化学发光免疫分析" or "化学发光微粒子免疫分析" or "化学发光免疫分析" or "酶联免疫吸附试验" or "ECLIA" or "CMIA" or "CLIA" or "ELISA" or "SARS‐CoV‐2核衣壳蛋白" or "SARS‐CoV‐2刺突蛋白" or "SARS‐CoV‐2刺突蛋白S1亚基和刺突蛋白S2亚基" or "SARS‐CoV‐2重组刺突蛋白S1/S2亚基") |
| **Pubmed** |
| ((COVID-19[Text Word]) OR (coronavirus disease 2019[Text Word]) OR (2019-nCoV[Text Word]) OR (2019 novel coronavirus[Text Word]) OR (SARS-CoV-2[Text Word]) OR (Anti-SARS-CoV-2[Text Word]) OR (severe acute respiratory syndrome coronavirus 2[Text Word])) AND ((COVID-19 diagnostic testing[Text Word]) OR (COVID-19 serological test[Text Word]) OR (“Serology”[MeSH Terms]) OR (“Antibodies”[MeSH Terms]) OR (“Immunoglobulins”[MeSH Terms]) OR (immunoglobulin[Text Word]) OR (“Immunoglobulin G”[MeSH Terms]) OR (IgG[Text Word]) OR (total antibody[Text Word]) OR (“Immunoglobulin A”[MeSH Terms]) OR (IgA[Text Word]) OR (“Enzyme-linked Immunosorbent Assay”[MeSH Terms]) OR (enzyme linked immunosorbent assay[Text Word]) OR (electrochemiluminescence immunoassays[Text Word]) OR (chemiluminescent microparticle immunoassays[Text Word]) OR (chemiluminescence immunoassays[Text Word]) OR (ECLIA[Text Word]) OR (CMIA[Text Word]) OR (CLIA[Text Word]) OR (ELISA[Text Word]) OR (EIA[Text Word]) OR (Abbott SARS-CoV-2 IgG[Text Word]) OR (Elecsys Anti-SARS-CoV-2 N[Text Word]) OR (Elecsys Anti-SARS-CoV-2 total[Text Word]) OR (Euroimmun Anti-SARS-CoV-2 IgA[Text Word]) OR (Euroimmun Anti-SARS-CoV-2 S1-IgG[Text Word]) OR (Euroimmun Anti-SARS-CoV-2 N-IgG[Text Word]) OR (LIAISON SARS-CoV-2 S1/S2 IgG[Text Word]) OR (nucleocapsid protein[Text Word]) OR (spike glycoproteins[Text Word]) OR (S1 subunit and S2 subunit[Text Word])) |
| **Embase** |
| #1 'COVID-19'/exp OR 'coronavirus disease 2019'/exp OR '2019-nCoV'/exp  #2 '2019 novel coronavirus*':ab,ti OR 'SARS-CoV-2':ab,ti OR 'Anti-SARS-CoV-2':ab,ti OR ' severe acute respiratory syndrome coronavirus 2*':ab,ti  #3 #1 OR #2  #4 'COVID-19 diagnostic testing'/exp OR 'COVID-19 serological test'/exp OR 'Serology'/exp OR 'Antibodies'/exp OR 'Immunoglobulins'/exp OR 'Immunoglobulin G'/exp OR 'IgG'/exp OR 'total antibody'/exp OR 'Immunoglobulin A'/exp OR 'IgA'/exp  #5 'Enzyme-linked Immunosorbent Assay'/exp OR 'enzyme linked immunosorbent assay'/exp OR 'electrochemiluminescence immunoassays'/exp OR 'chemiluminescent microparticle immunoassays'/exp OR 'chemiluminescence immunoassays'/exp OR 'ECLIA'/exp OR 'CMIA'/exp OR 'CLIA'/exp OR 'ELISA'/exp OR 'EIA'/exp  #6 'Abbott SARS-CoV-2 IgG':ab,ti OR 'Elecsys Anti-SARS-CoV-2 N':ab,ti OR 'Elecsys Anti-SARS-CoV-2 total':ab,ti OR 'Euroimmun Anti-SARS-CoV-2 IgA':ab,ti OR 'Euroimmun Anti-SARS-CoV-2 S1-IgG':ab,ti OR 'Euroimmun Anti-SARS-CoV-2 N-IgG':ab,ti OR 'LIAISON SARS-CoV-2 S1/S2 IgG':ab,ti  #7 #4 OR #5  #8 'nucleocapsid protein'/exp OR 'spike glycoproteins'/exp OR 'S1 subunit and S2 subunit'/exp  #9 #6 OR #8  #10 #1 AND #4 AND #8  #11 #2 AND #4 AND #8 |
| **The Cochrane library** |
| 1.MeSH descriptor: [COVID-19] explode all trees  2.MeSH descriptor: [coronavirus disease 2019] explode all trees  3.MeSH descriptor: [2019-nCoV] explode all trees  4.MeSH descriptor: [2019 novel coronavirus] explode all trees  5.MeSH descriptor: [SARS-CoV-2] explode all trees  6.MeSH descriptor: [severe acute respiratory syndrome coronavirus 2] explode all trees  7.MeSH descriptor: [Anti-SARS-CoV-2] explode all trees  8.(COVID-19 diagnostic testing* OR COVID-19 serological test OR Serology OR Antibodies*):ti,ab,kw AND (SARS-CoV-2):ti,ab,kw  9.#1 OR #2 OR #3 OR #4 OR #5 OR #6 OR #7 OR #8  10.MeSH descriptor: [Immunoglobulins] explode all trees  11.MeSH descriptor: [immunoglobulin] explode all trees  12.MeSH descriptor: [Immunoglobulin G] explode all trees  13.MeSH descriptor: [IgG] explode all trees  14.MeSH descriptor: [total antibody] explode all trees  15.MeSH descriptor: [Immunoglobulin A] explode all trees  16.MeSH descriptor: [IgA] explode all trees  17.MeSH descriptor: [Nurseries, Infant] explode all trees  18.(Abbott SARS-CoV-2 IgG* OR Elecsys Anti-SARS-CoV-2 N* OR Elecsys Anti-SARS-CoV-2 total* OR Euroimmun Anti-SARS-CoV-2 IgA* OR Euroimmun Anti-SARS-CoV-2 S1-IgG* OR Euroimmun Anti-SARS-CoV-2 N-IgG* OR LIAISON SARS-CoV-2 S1/S2 IgG*):ti,ab,kw  19.#10 OR #11 OR #12 OR #13 OR #14 OR #15 OR #16 OR #17  20.#9 AND #18  21.(Enzyme-linked Immunosorbent Assay OR enzyme linked immunosorbent assay OR electrochemiluminescence immunoassays OR chemiluminescent microparticle immunoassays OR chemiluminescence immunoassays OR ECLIA OR CMIA OR CLIA OR ELISA OR EIA):ti,ab,kw  22.MeSH descriptor: [nucleocapsid protein] OR [spike glycoproteins] OR[S1 subunit and S2 subunit] explode all trees  23.#21 OR #22  24.#18 AND #22 with Publication Year from 2019 to 2024 |
| **Web of Science** |
| 1: TI=(COVID-19) OR TI=(coronavirus disease 2019) OR TI=(2019-nCoV) OR TI=(2019 novel coronavirus) OR TI=(SARS-CoV-2) OR TI=(severe acute respiratory syndrome coronavirus 2) OR TI=(COVID-19 diagnostic testing) OR TI=(COVID-19 serological test) OR TI=(nucleocapsid protein) OR TI=(spike glycoproteins) OR TI=(S1 subunit and S2 subunit) and Anti-SARS-CoV-2 antibody (Exclude – Database) Timespan: 2019-10-01 to 2024-06-25  2: TI=(Abbott SARS-CoV-2 IgG) TI=(Elecsys Anti-SARS-CoV-2 N) TI=(Elecsys Anti-SARS-CoV-2 total) TI=(Euroimmun Anti-SARS-CoV-2 IgA) TI=(Euroimmun Anti-SARS-CoV-2 S1-IgG) TI=(Euroimmun Anti-SARS-CoV-2 N-IgG) TI=(LIAISON SARS-CoV-2 S1/S2 IgG) and COVID-19 (Exclude – Database) Timespan: 2019-10-01 to 2024-06-25  3: #2 AND #1 (Exclude – Database) Timespan: 2019-10-01 to 2024-06-25 Results: |
