# Supplementary material for: An Indirect Comparison of Diagnostic Accuracy for Seven Different SARS‐CoV‐2 Serological Assays: A Meta‐Analysis and Adjusted Indirect Comparison of Diagnostic Test Accuracy
Source: Influenza Other Respir Viruses. 2025 Sep 9;19(9):e70155. doi: 10.1111/irv.70155 (PMC12418076; doi:10.1111/irv.70155)
Supplement: Supplementary file 3 — Appendix S3: Table S1 Characteristics of the included studies. (DOC) [file IRV-19-e70155-s002.doc]

**Appendix 3. Table S1**  Characteristics of the included studies

| **Author** | **Year** | **Method** | **Manufacturer/ platform** | **Assay** | **Antigen** | **Antibody type** | **COVID-19 patient samples(*n*)** | **Controls**  **(*n*)** |
| --- | --- | --- | --- | --- | --- | --- | --- | --- |
|
| A Bown^[11]^ | 2020 | CLIA | Abbott Architect i2000sr | Abbott SARS-CoV-2 IgG | N | IgG | 536 | 976 |
|  |  | CLIA | LIAISON XL analyze | LIAISON SARS-CoV-2 S1/S2 IgG | S1 and S2 | IgG | 536 | 976 |
|  |  | ECLIA | Roche Cobas e411 | Elecsys Anti-SARS-CoV-2 N | N | Total Antibody | 536 | 976 |
| Andrey DO^[12]^ | 2021 | ELISA | LIAISON XL analyzer | LIAISON anti‐SARS‐CoV‐2 S1/S2 IgG | S1 and S2 | IgG | 172 | 185 |
|  |  | ELISA | Agility EUROIMMUN | Euroimmun Anti-SARS-CoV-2 S1-IgG | S1 | IgG | 172 | 185 |
|  |  | ECLIA | Roche Cobas e801 | Elecsys Anti-SARS-CoV-2 N | N | Total Antibody | 172 | 185 |
| Beavis KG^[13]^ | 2020 | ELISA | NA | Euroimmun Anti-SARS-CoV-2 S1-IgG | S1 | IgG | 82 | 86 |
|  |  | ELISA | NA | Euroimmun Anti-SARS-CoV-2 IgA | S1 | IgA | 82 | 86 |
| Chan CW^[14]^ | 2020 | ECLIA | Roche Cobas e602 | Elecsys Anti-SARS-CoV-2 N | N | Total Antibody | 78 | 53 |
| Chansaenroj J^[15]^ | 2021 | CMIA | Abbott Architect i1000sr | Abbott SARS-CoV-2 IgG | N | IgG | 245 | 130 |
|  |  | ELISA | Analyzer I-2P machine | Euroimmun Anti-SARS-CoV-2 S1-IgG | S1 | IgG | 245 | 130 |
|  |  | ELISA | Analyzer I-2P machine | Euroimmun Anti-SARS-CoV-2 IgA | S1 | IgA | 245 | 130 |
| Chen SY^[16]^ | 2020 | ECLIA | Roche Cobas e analyzers (e 411, e 601, and e 602) | Elecsys Anti-SARS-CoV-2 N | N | Total Antibody | 346 | 194 |
|  |  | CMIA | Abbott Architect i System (i2000sr and i1000sr) | Abbott SARS-CoV-2 IgG | N | IgG | 346 | 194 |
| Chiereghin A^[17]^ | 2020 | CLIA | LIAISON XL analyze | LIAISON SARS-CoV-2 S1/S2 IgG | S1 and S2 | IgG | 207 | 130 |
|  |  | ECLIA | Roche Cobas e801 | Elecsys Anti-SARS-CoV-2 N | N | Total Antibody | 207 | 130 |
|  |  | ELISA | Euroimmun Analyzer 1 | Euroimmun Anti-SARS-CoV-2 IgA | S1 | IgA | 185 | 109 |
| Chua KYL^[18]^ | 2020 | ECLIA | Roche Cobas e801 | Elecsys Anti-SARS-CoV-2 N | N | Total Antibody | 86 | 95 |
|  |  | CLIA | LIAISON XL analyze | LIAISON SARS-CoV-2 S1/S2 IgG | S1 and S2 | IgG | 86 | 95 |
| Davidson N^[19]^ | 2020 | ELISA | NA | Euroimmun Anti-SARS-CoV-2 S1-IgG | S1 | IgG | 71 | 138 |
|  |  | ELISA | NA | Euroimmun Anti-SARS-CoV-2 IgA | S1 | IgA | 71 | 138 |
| Eberhardt KA^[20]^ | 2021 | ELISA | Euroimmun Analyzer I | Euroimmun Anti-SARS-CoV-2 S1-IgG | S1 | IgG | 363 | 227 |
|  |  | CLIA | LIAISON XL analyze | LIAISON SARS-CoV-2 S1/S2 IgG | S1 and S2 | IgG | 363 | 227 |
|  |  | ECLIA | Roche Cobas 8000 | Elecsys Anti-SARS-CoV-2 N | N | Total Antibody | 363 | 227 |
|  |  | CLIA | Abbott Alinity | Abbott SARS-CoV-2 IgG | N | IgG | 363 | 227 |
| Egger M^[21]^ | 2020 | ECLIA | Roche Cobas e801 | Elecsys Anti-SARS-CoV-2 N | N | Total Antibody | 104 | 200 |
| Ekelund O^[22]^ | 2021 | CMIA | Abbott Architect i2000sr | Abbott SARS-CoV-2 IgG | N | IgG | 152 | 150 |
|  |  | ECLIA | Roche Cobas e801 | Elecsys Anti-SARS-CoV-2 N | N | Total Antibody | 152 | 150 |
|  |  | CLIA | LIAISON XL analyze | LIAISON SARS-CoV-2 S1/S2 IgG | S1 and S2 | IgG | 145 | 148 |
| Emmerich P^[23]^ | 2021 | ELISA | NA | Euroimmun Anti-SARS-CoV-2 S1-IgG | S1 | IgG | 129 | 54 |
|  |  | ELISA | NA | Euroimmun Anti-SARS-CoV-2 IgA | S1 | IgA | 129 | 54 |
|  |  | ELISA | NA | Euroimmun Anti-SARS-CoV-2 IgM | N | IgM | 129 | 54 |
|  |  | ELISA | NA | Euroimmun Anti-SARS-CoV-2 N-IgG | N | IgG | 129 | 54 |
| Favresse J^[24]^ | 2021 | ELISA | EUROIMMUN Analyzer I‐2P | Euroimmun Anti-SARS-CoV-2 N-IgG | N | IgG | 87 | 141 |
|  |  | ECLIA | Roche Cobas e801 | Elecsys Anti-SARS-CoV-2 N | N | Total Antibody | 87 | 141 |
|  |  | ELISA | LIAISON XL analyzer | LIAISON anti‐SARS‐CoV‐2 S1/S2 IgG | S1 and S2 | IgG | 87 | 141 |

**Table S1** (continued)

| **Author** | **Year** | **Method** | **Manufacturer/ platform** | **Assay** | **Antigen** | **Antibody type** | **COVID-19 patient samples(*n*)** | **Controls**  **(*n*)** |
| --- | --- | --- | --- | --- | --- | --- | --- | --- |
|
| Fischer PU^[25]^ | 2021 | ELISA | NA | Euroimmun Anti-SARS-CoV-2 S1-IgG | S1 | IgG | 290 | 168 |
|  |  | CMIA | Abbott Architect | Abbott SARS-CoV-2 IgG | N | IgG | 265 | 156 |
| Harritshøj LH^[26]^ | 2021 | CLIA | Roche Cobas e601 | Elecsys Anti–SARS-CoV-2 total | N | Total Antibody | 150 | 218 |
|  |  | CMIA | Abbott Alinity | Abbott SARS-CoV-2 IgG | N | IgG | 150 | 600 |
|  |  | CMIA | Abbott Architect | Abbott SARS-CoV-2 IgG | N | IgG | 150 | 603 |
|  |  | CLIA | LIAISON XL analyze | LIAISON SARS-CoV-2 S1/S2 IgG | S1 and S2 | IgG | 150 | 1388 |
|  |  | ELISA | Euroimmun Analyzer 1 | Euroimmun Anti-SARS-CoV-2 S1-IgG | S1 | IgG | 150 | 599 |
| Heffernan E^[27]^ | 2021 | CLIA | Roche Cobas e600 | Elecsys Anti–SARS-CoV-2 total | N | Total Antibody | 137 | 100 |
|  |  | ELISA | Dynex DS2 | Euroimmun Anti-SARS-CoV-2 S1-IgG | S1 | IgG | 137 | 100 |
|  |  | ELISA | Dynex DS2 | Euroimmun Anti-SARS-CoV-2 N-IgG | N | IgG | 137 | 100 |
| Herroelen PH^[28]^ | 2020 | CLIA | LIAISON XL analyze | LIAISON SARS-CoV-2 S1/S2 IgG | S1 and S2 | IgG | 168 | 56 |
|  |  | ELISA | Bio-Rad Version EIA 0_16 | Euroimmun Anti-SARS-CoV-2 IgA | S1 | IgA | 169 | 56 |
|  |  | ELISA | Bio-Rad Version EIA 0_16 | Euroimmun Anti-SARS-CoV-2 S1-IgG | S1 | IgG | 169 | 56 |
|  |  | ELISA | Bio-Rad Version EIA 0_16 | Euroimmun Anti-SARS-CoV-2 N-IgG | N | IgG | 170 | 56 |
|  |  | CLIA | Roche Cobas e601 | Elecsys Anti–SARS-CoV-2 total | N | Total Antibody | 170 | 56 |
| Hörber S^[29]a^ | 2020 | ECLIA | Roche Cobas e411 | Elecsys Anti-SARS-CoV-2 N | N | Total Antibody | 186 | 123 |
|  |  | ELISA | BEP 2000 Advance | Euroimmun Anti-SARS-CoV-2 S1-IgG | S1 | IgG | 186 | 123 |
| Horn MP^[30]^ | 2022 | ELISA | DYNEX DSX | Euroimmun Anti-SARS-CoV-2 S1-IgG | S1 | IgG | 195 | 3463 |
|  |  | ECLIA | Roche Cobas 8000 | Elecsys Anti-SARS-CoV-2 N | N | Total Antibody | 189 | 3441 |
|  |  | CLIA | Abbott Architect i2000sr | Abbott SARS-CoV-2 IgG | N | IgG | 189 | 3441 |
|  |  | CLIA | LIAISON XL analyze | LIAISON SARS-CoV-2 S1/S2 IgG | S1 and S2 | IgG | 189 | 3441 |
| Ige F^[31]^ | 2021 | ELISA | NA | Euroimmun Anti-SARS-CoV-2 N-IgG | N | IgG | 95 | 99 |
|  |  | ELISA | NA | Euroimmun Anti-SARS-CoV-2 S1-IgG | S1 | IgG | 96 | 99 |
| Ikegami S^[32]^ | 2021 | ECLIA | Roche Cobas e analyzers | Elecsys Anti-SARS-CoV-2 N | N | Total Antibody | 97 | 100 |
|  |  | CLIA | LIAISON XL analyze | LIAISON SARS-CoV-2 S1/S2 IgG | S1 and S2 | IgG | 97 | 100 |
| Irsara C^[33]^ | 2021 | CLIA | Roche Cobas e602 | Elecsys Anti–SARS-CoV-2 total | N | Total Antibody | 230 | 341 |
|  |  | CMIA | Abbott Architect i2000sr | Abbott SARS-CoV-2 IgG | N | IgG | 228 | 298 |
| Jung K^[34]^ | 2021 | CMIA | Abbott Architect i System | Abbott SARS-CoV-2 IgG II Quant | S1-RBD | IgG | 173 | 151 |
|  |  | CLIA | LIAISON XL analyze | LIAISON SARS-CoV-2 S1/S2 IgG | S1 and S2 | IgG | 173 | 151 |
|  |  | ECLIA | Roche Cobas e601 | Elecsys Anti-SARS-CoV-2 N | N | Total Antibody | 173 | 151 |
| Kubota K^[35]^ | 2021 | CLIA | Abbott Architect i2000sr | Abbott SARS-CoV-2 IgG | N | IgG | 66 | 148 |
|  |  | ECLIA | Roche Cobas e801 | Elecsys Anti-SARS-CoV-2 N | N | Total Antibody | 66 | 148 |
| Kundu D^[36]^ | 2022 | ECLIA | Roche Cobas e401 | Elecsys Anti-SARS-CoV-2 N | N | Total Antibody | 153 | 150 |
|  |  | CLIA | Abbott Architect i2000sr | Abbott SARS-CoV-2 IgG | N | IgG | 153 | 150 |
|  |  | CLIA | LIAISON XL analyze | LIAISON SARS-CoV-2 S1/S2 IgG | S1 and S2 | IgG | 153 | 150 |

**Table S1 (continued)**

| **Author** | **Year** | **Method** | **Manufacturer/ platform** | **Assay** | **Antigen** | **Antibody type** | **COVID-19 patient samples(*n*)** | **Controls**  **(*n*)** |
| --- | --- | --- | --- | --- | --- | --- | --- | --- |
|  |  |  |  |  |  |  |  |  |
| Lau CS^[37]^ | 2020 | CMIA | Abbott Architect i2000sr | Abbott SARS-CoV-2 IgG | N | IgG | 386 | 980 |
| Lester SN^[38]^ | 2024 | CMIA | Abbott Architect i System (i2000sr and i1000sr) | Abbott SARS-CoV-2 IgG | N | IgG | 87 | 117 |
| Mafi S^[39]^ | 2023 | CLIA | LIAISON XL analyze | LIAISON SARS-CoV-2 S1/S2 IgG | S1 and S2 | IgG | 110 | 120 |
|  |  | CMIA | Abbott Architect i System | Abbott SARS-CoV-2 IgG | N | IgG | 110 | 120 |
| Manalac J^[40]^ | 2020 | CMIA | Abbott Architect i2000sr | Abbott SARS-CoV-2 IgG | N | IgG | 97 | 847 |
|  |  | ELISA | EUROLab Workstation | Euroimmun Anti-SARS-CoV-2 S1-IgG | S1 | IgG | 97 | 847 |
| Merrill AE^[41]^ | 2020 | ECLIA | Roche Cobas e602 | Elecsys Anti-SARS-CoV-2 N | N | Total Antibody | 54 | 174 |
|  |  | CLIA | LIAISON XL analyze | LIAISON SARS-CoV-2 S1/S2 IgG | S1 and S2 | IgG | 54 | 174 |
| Montesinos I^[42]^ | 2020 | ELISA | ETI-MAX 3000 | Euroimmun Anti-SARS-CoV-2 S1-IgG | S1 | IgG | 128 | 72 |
|  |  | ELISA | Euroimmun Analyzer 1 | Euroimmun Anti-SARS-CoV-2 IgA | S1 | IgA | 128 | 72 |
| Muench P^[43]^ | 2020 | ECLIA | Roche Cobas e analyzers | Elecsys Anti-SARS-CoV-2 N | N | Total Antibody | 496 | 10453 |
| Mylemans M^[44]^ | 2021 | ELISA | Inova QUANTA-Lyser 2 | Euroimmun Anti-SARS-CoV-2 N-IgG | N | IgG | 81 | 85 |
|  |  | CLIA | Roche Cobas e601 | Elecsys Anti–SARS-CoV-2 total | N | Total Antibody | 81 | 85 |
|  |  | ELISA | Inova QUANTA-Lyser 2 | Euroimmun Anti-SARS-CoV-2 S1-IgG | S1 | IgG | 81 | 85 |
|  |  | CLIA | LIAISON XL analyze | LIAISON SARS-CoV-2 S1/S2 IgG | S1 and S2 | IgG | 81 | 85 |
| Naaber P^[45]^ | 2020 | CLIA | LIAISON XL analyze | LIAISON SARS-CoV-2 S1/S2 IgG | S1 and S2 | IgG | 97 | 100 |
|  |  | ELISA | Agility EUROIMMUN | Euroimmun Anti-SARS-CoV-2 S1-IgG | S1 | IgG | 97 | 100 |
|  |  | CMIA | Abbott Architect i2000sr | Abbott SARS-CoV-2 IgG | N | IgG | 97 | 100 |
|  |  | ECLIA | Roche Cobas e411 | Elecsys Anti-SARS-CoV-2 N | N | Total Antibody | 97 | 100 |
| Nedelcu I^[46]^ | 2021 | ECLIA | Roche Cobas e411 | Elecsys Anti-SARS-CoV-2 N | N | Total Antibody | 559 | 161 |
|  |  | CLIA | LIAISON XL analyze | LIAISON SARS-CoV-2 S1/S2 IgG | S1 and S2 | IgG | 559 | 161 |
|  |  | CMIA | Abbott Architect i2000sr | Abbott SARS-CoV-2 IgG | N | IgG | 528 | 161 |
| Nicholson S^[47]^ | 2021 | ELISA | NA | Euroimmun Anti-SARS-CoV-2 S1-IgG | S1 | IgG | 147 | 179 |
|  |  | ELISA | NA | Euroimmun Anti-SARS-CoV-2 IgA | S1 | IgA | 147 | 179 |
|  |  | ELISA | NA | Euroimmun Anti-SARS-CoV-2 N-IgG | N | IgG | 146 | 191 |
| Olbrich L^[48]^ | 2021 | ELISA | Euroanalyser-1 robot | Euroimmun Anti-SARS-CoV-2 S1-IgG | S1 | IgG | 193 | 1073 |
|  |  | ELISA | Euroanalyser-1 robot | Euroimmun Anti-SARS-CoV-2 IgA | S1 | IgA | 193 | 1073 |
|  |  | ECLIA | Roche Cobas 400-e411 and/or 8000-e801 | Elecsys Anti-SARS-CoV-2 N | N | Total Antibody | 193 | 1073 |
| Padoan A^[49]^ | 2020 | ECLIA | Roche Cobas e602 | Elecsys Anti-SARS-CoV-2 N | N | Total Antibody | 130 | 42 |
|  |  | CMIA | Abbott Architect | Abbott SARS-CoV-2 IgG | N | IgG | 130 | 54 |
| Parai D^[50]^ | 2021 | CMIA | Abbott Architect i2000sr | Abbott SARS-CoV-2 IgG | N | IgG | 594 | 100 |
|  |  | ECLIA | Roche Cobas e411 | Elecsys Anti-SARS-CoV-2 N | N | Total Antibody | 594 | 100 |
| Pérez-García F^[51]^ | 2021 | CLIA | Roche Cobas e analyzers | Elecsys Anti–SARS-CoV-2 total | N | Total Antibody | 50 | 60 |
| Rikhtegaran Tehrani Z^[52]^ | 2020 | ELISA | NA | Euroimmun Anti-SARS-CoV-2 S1-IgG | S1 | IgG | 99 | 298 |
|  |  | ELISA | NA | Euroimmun Anti-SARS-CoV-2 IgA | S1 | IgA | 100 | 284 |

**Table S1 (continued)**

| **Author** | **Year** | **Method** | **Manufacturer/ platform** | **Assay** | **Antigen** | **Antibody type** | **COVID-19 patient samples(*n*)** | **Controls**  **(*n*)** |
| --- | --- | --- | --- | --- | --- | --- | --- | --- |
|
| Piec I^[53]^ | 2021 | ELISA | Manual | Euroimmun Anti-SARS-CoV-2 S1-IgG | S1 | IgG | 43 | 152 |
|  |  | CMIA | Abbott Alinity | Abbott SARS-CoV-2 IgG | N | IgG | 43 | 152 |
|  |  | CLIA | LIAISON XL analyzer | LIAISON SARS-CoV-2 S1/S2 IgG | S1 and S2 | IgG | 42 | 152 |
|  |  | CMIA | Abbott Architect | Abbott SARS-CoV-2 IgG | N | IgG | 94 | 99 |
| Sekirov I^[54]^ | 2021 | CLIA | LIAISON XL analyze | LIAISON SARS-CoV-2 S1/S2 IgG | S1 and S2 | IgG | 42 | 65 |
|  |  | CLIA | Abbott Architect | Abbott SARS-CoV-2 IgG | N | IgG | 42 | 65 |
|  |  | CLIA | Roche Cobas e601 | Elecsys Anti–SARS-CoV-2 total | N | Total Antibody | 42 | 65 |
| Serre-Miranda C^[55]^ | 2021 | CLIA | Abbott Architect i2000sr | Abbott SARS-CoV-2 IgG | N | IgG | 120 | 39 |
|  |  | ELISA | NA | Euroimmun Anti-SARS-CoV-2 S1-IgG | S1 | IgG | 126 | 38 |
|  |  | ELISA | NA | Euroimmun Anti-SARS-CoV-2 IgA | S1 | IgA | 126 | 38 |
| Syre H^[56]^ | 2022 | CLIA | LIAISON XL analyze | LIAISON SARS-CoV-2 S1/S2 IgG | S1 and S2 | IgG | 211 | 320 |
|  |  | CLIA | Abbott Alinity | Abbott SARS-CoV-2 IgG | N | IgG | 211 | 320 |
| Tan SS^[57]^ | 2020 | CLIA | Abbott Architect i4000sr | Abbott SARS-CoV-2 IgG | N | IgG | 173 | 163 |
|  |  | ECLIA | Roche Cobas e411 | Elecsys Anti-SARS-CoV-2 N | N | Total Antibody | 173 | 163 |
| Tanis J^[58]b^ | 2021 | CLIA | Abbott Architect i System | Abbott SARS-CoV-2 IgG | N | IgG | 52 | 36 |
|  |  | CLIA | LIAISON XL analyze | LIAISON SARS-CoV-2 S1/S2 IgG | S1 and S2 | IgG | 52 | 35 |
|  |  | ELISA | EUROLab Workstation | Euroimmun Anti-SARS-CoV-2 S1-IgG | S1 | IgG | 49 | 37 |
|  |  | ECLIA | Roche Cobas e801 | Elecsys Anti-SARS-CoV-2 N | N | Total Antibody | 52 | 36 |
| Theel ES^[59]^ | 2020 | ELISA | Dynex Agility | Euroimmun Anti-SARS-CoV-2 S1-IgG | S1 | IgG | 61 | 149 |
|  |  | CMIA | Abbott Architect i2000sr | Abbott SARS-CoV-2 IgG | N | IgG | 61 | 149 |
| Tolan NV^[60]^ | 2023 | CMIA | Abbott Architect i System | Abbott SARS-CoV-2 IgG | N | IgG | 124 | 59 |
|  |  | CLIA | LIAISON XL analyze | LIAISON SARS-CoV-2 S1/S2 IgG | S1 and S2 | IgG | 76 | 75 |
|  |  | ELISA | NA | Euroimmun Anti-SARS-CoV-2 S1-IgG | S1 | IgG | 61 | 57 |
|  |  | ELISA | NA | Euroimmun Anti-SARS-CoV-2 IgA | S1 | IgA | 60 | 52 |
|  |  | ECLIA | Roche Cobas e analyzers | Elecsys Anti-SARS-CoV-2 N | N | Total Antibody | 150 | 88 |
| Tré-Hardy M^[61]^ | 2020 | CLIA | LIAISON XL analyzer | LIAISON SARS-CoV-2 S1/S2 IgG | S1 and S2 | IgG | 44 | 81 |
|  |  | ELISA | ETI-MAX 3000 | Euroimmun Anti-SARS-CoV-2 S1-IgG | S1 | IgG | 44 | 81 |
| Van Elslande J^[62]^ | 2020 | ECLIA | Roche Cobas e802 | Elecsys Anti-SARS-CoV-2 N | N | Total Antibody | 223 | 113 |
|  |  | CLIA | Abbott Architect i2000sr | Abbott SARS-CoV-2 IgG | N | IgG | 223 | 113 |
|  |  | ELISA | Siemens BEP III | Euroimmun Anti-SARS-CoV-2 N-IgG | N | IgG | 223 | 113 |
|  |  | ELISA | Siemens BEP III | Euroimmun Anti-SARS-CoV-2 S1-IgG | S1 | IgG | 223 | 113 |
|  |  | CLIA | LIAISON XL analyzer | LIAISON SARS-CoV-2 S1/S2 IgG | S1 and S2 | IgG | 223 | 113 |
| Velay A^[63]^ | 2020 | ELISA | NA | Euroimmun Anti-SARS-CoV-2 S1-IgG | S1 | IgG | 198 | 100 |
|  |  | ELISA | NA | Euroimmun Anti-SARS-CoV-2 IgA | S1 | IgA | 198 | 100 |
| Ward MD^[64]^ | 2021 | CMIA | Abbott Architect | Abbott SARS-CoV-2 IgG | N | IgG | 112 | 2030 |
|  |  | CLIA | Roche Cobas e analyzers | Elecsys Anti–SARS-CoV-2 total | N | Total Antibody | 112 | 2030 |

**Table S1** (continued)

| **Author** | **Year** | **Method** | **Manufacturer/ platform** | **Assay** | **Antigen** | **Antibody type** | **COVID-19 patient samples(*n*)** | **Controls**  **(*n*)** |
| --- | --- | --- | --- | --- | --- | --- | --- | --- |
|
| Wolff F^[65]^ | 2020 | ECLIA | Roche Cobas e801 | Elecsys Anti-SARS-CoV-2 N | N | Total Antibody | 111 | 96 |
|  |  | CLIA | LIAISON XL analyze | LIAISON SARS-CoV-2 S1/S2 IgG | S1 and S2 | IgG | 111 | 96 |
|  |  | ELISA | ETI-MAX 3000 | Euroimmun Anti-SARS-CoV-2 S1-IgG | S1 | IgG | 111 | 96 |
|  |  | ELISA | ETI-MAX 3000 | Euroimmun Anti-SARS-CoV-2 IgA | S1 | IgA | 111 | 96 |
| Zilla M^[66]^ | 2021 | CLIA | Roche Cobas e400 | Elecsys Anti–SARS-CoV-2 total | N | Total Antibody | 154 | 184 |
|  |  | ELISA | Bio-Rad | Euroimmun Anti-SARS-CoV-2 S1-IgG | S1 | IgG | 154 | 184 |
|  |  | ELISA | Bio-Rad | Euroimmun Anti-SARS-CoV-2 IgA | S1 | IgA | 154 | 184 |
| Zonneveld R^[67]^ | 2021 | CLIA | LIAISON XL analyze | LIAISON SARS-CoV-2 S1/S2 IgG | S1 and S2 | IgG | 126 | 158 |
|  |  | ECLIA | Roche Cobas e601 | Elecsys Anti-SARS-CoV-2 N | N | Total Antibody | 126 | 158 |
|  |  | CMIA | Abbott Architect i2000sr | Abbott SARS-CoV-2 IgG | N | IgG | 126 | 158 |

Abbreviations: CLIA, chemiluminescent immunoassay; CMIA, Chemiluminescence microparticle immunoassay; ECLIA, Electrochemiluminescent immunoassay; ELISA, Enzyme immunoassay; N: Nucleocapsid antigen; S1: S1 domain of viral spike protein; S1/S2: Recombinant S1 and S2 antigens; NA, not specifed.

^a^If borderline results were considered positive.

^b^The number of inpatient group patients providing samples collected ≥15 days post-symptom onset.
