# Supplementary material for: An Indirect Comparison of Diagnostic Accuracy for Seven Different SARS‐CoV‐2 Serological Assays: A Meta‐Analysis and Adjusted Indirect Comparison of Diagnostic Test Accuracy
Source: Influenza Other Respir Viruses. 2025 Sep 9;19(9):e70155. doi: 10.1111/irv.70155 (PMC12418076; doi:10.1111/irv.70155)
Supplement: Supplementary file 4 — Appendix S4: Table S2 Quality assessment of QUADAS‐2 tool with explanation. (DOC) [file IRV-19-e70155-s006.doc]

**Appendix 4.** Table S2 Quality assessment of QUADAS-2 tool with explanation

| **Domain and Description** | **Signaling question** | **Explanation** |
| --- | --- | --- |
| **1. Patient selection** - describe methods of patient selection: Describe included patients (prior testing, presentation, intended use of index test and setting) | Signaling question 1: Was a consecutive or random sample of patients enrolled? | The question 1 was answered as “no” for Chen^[16]^, for the reason that the COVID-19 patient serum samples were collected from six participating hospitals. Other 56 articles were answered as “yes”. |
|  | Signaling question 2: Was a case-control design avoided? | The question 2 was answered as “no” for Chiereghin^[17]^, for the reason that the study was a retrospective case-control study. Other 56 articles were answered as “yes”. |
|  | Signaling question 3: were the inclusion/exclusion criteria specified? | The question 3 was answered as “no” for Ikegami^[32]^, Naaber^[45]^ and Nedelcu^[46]^, for the reason that the specificity was evaluated using serum samples from healthy persons before COVID-19 pandemic as negative controls. Other 54 articles were answered as “yes”. |
| **Risk of bias** | Could the selection of patients have introduced bias? | Any one of the three questions was answered as “no”, then the level of risk of bias was judged as “high”. |
| **Applicability concern** | Are there concerns that the included patients do not match the review question? | Studies included samples from asymptomatic individuals who were screened positive for SARS-CoV-2 nucleic acid without any symptoms, which were judged as “high”.  Studies were judged as “unclear” in which serum samples were collected from recovered COVID-19 patients. |
| **2. Index test** - describe the index test and how it was conducted and interpreted | Signaling question 1: Were the index test results interpreted without knowledge of the results of the reference standard? | The question 1 was answered as “no” among the 57 articles on account for the serums samples obtained for serologic testing and the serological assays were evaluated not in blind. |
|  | Signaling question 2: If a threshold was used, was it prespecified? | The question 2 was answered as “yes” among the 57 articles for the reason that all the assays and the test systems were evaluated, using the cut-off provided by the manufacturer. |
| **Risk of bias** | Could methods used to conduct or interpret the index test have introduced bias? | Any one of the two questions was answered as “no”, then the level of risk of bias was judged as “unclear”. Therefore all 57 articles were judged as “unclear”. |
| **Applicability concern** | Are there concerns that the index test, its conduct, or interpretation differ from the review question? | All serologic testing and the serological assays were evaluated and all the test systems, theirs conduct, or interpretation were evaluated. Therefore all 57 articles were judged as “low”. |
| **3. Reference standard** - describe the reference standard and how it was conducted and interpreted | Signaling question 1: Is the reference standard likely to correctly classify the target condition? | Studies included persons who had been diagnosed COVID-19 by positive SARS-CoV-2 RT-PCR regardless of clinical symptoms or in which the serum samples were collected from recovered COVID-19 patients were judged as “unclear”. |
|  | Signaling question 2: Was the reference standard results interpreted without knowledge of the results of the index test? | All 57 articles evaluated the diagnostic accuracy of serological assays using the reference standard RT-PCR technique as the gold standard without knowledge of the results of serological assays. Therefore the question 2 was answered as “yes” among the 57 articles. |
| **Risk of bias** | Could methods used to conduct or interpret the reference standard have introduced bias? | The question 1 was answered as “unclear”, then the level of risk of bias was judged as “unclear”. |
| **Applicability concern** | Are there concerns the target condition as defined by the reference standard does not match the question? | Studies included samples from asymptomatic individuals who were screened positive for SARS-CoV-2 nucleic acid without any symptoms, which were judged as “high”.  Studies were judged as “unclear” in which serum samples were collected from recovered COVID-19 patients. |
| **4. Flow and timing** - describe the time interval and any interventions between index test (s) and reference standard | Signaling question 1: Was there an appropriate interval between index test and reference standard? | We set the time range ≥7 days as the appropriate time interval. If the time between post-first RT-PCR positive result and the serum samples collected from COVID-19 patients within 7 days, the question 1 was answered as “no”. |
|  | Signaling question 2: Did all patients receive the same reference standard? | If the studies in which different protocols and RT-PCR reagents were employed, the question 2 was answered as “no”. |
|  | Signaling question 3: Were all patients included in the analysis? | If the studies in which serum samples were found to have insufficient sample volumes and were excluded from the validation, the question 3 was answered as “no”. |
| **Risk of bias** | Could the patient flow have introduced bias? | Two or three questions were answered as “no”, therefore the level of risk of bias was judged as “high”. |
