# Supplementary material for: An Indirect Comparison of Diagnostic Accuracy for Seven Different SARS‐CoV‐2 Serological Assays: A Meta‐Analysis and Adjusted Indirect Comparison of Diagnostic Test Accuracy
Source: Influenza Other Respir Viruses. 2025 Sep 9;19(9):e70155. doi: 10.1111/irv.70155 (PMC12418076; doi:10.1111/irv.70155)
Supplement: Supplementary file 5 — Appendix S5: Sensitivity analysis for Abbott SARS‐CoV‐2 IgG, Elecsys Anti‐SARS‐CoV‐2 N, Euroimmun Anti‐SARS‐CoV‐2 IgA and Euroimmun Anti‐SARS‐CoV‐2 N‐IgG. (DOC) [file IRV-19-e70155-s001.doc]

**Appendix 5.** Sensitivity analysis for Abbott SARS-CoV-2 IgG, Elecsys Anti-SARS-CoV-2 N, Euroimmun Anti-SARS-CoV-2 IgA and Euroimmun Anti-SARS-CoV-2 N-IgG


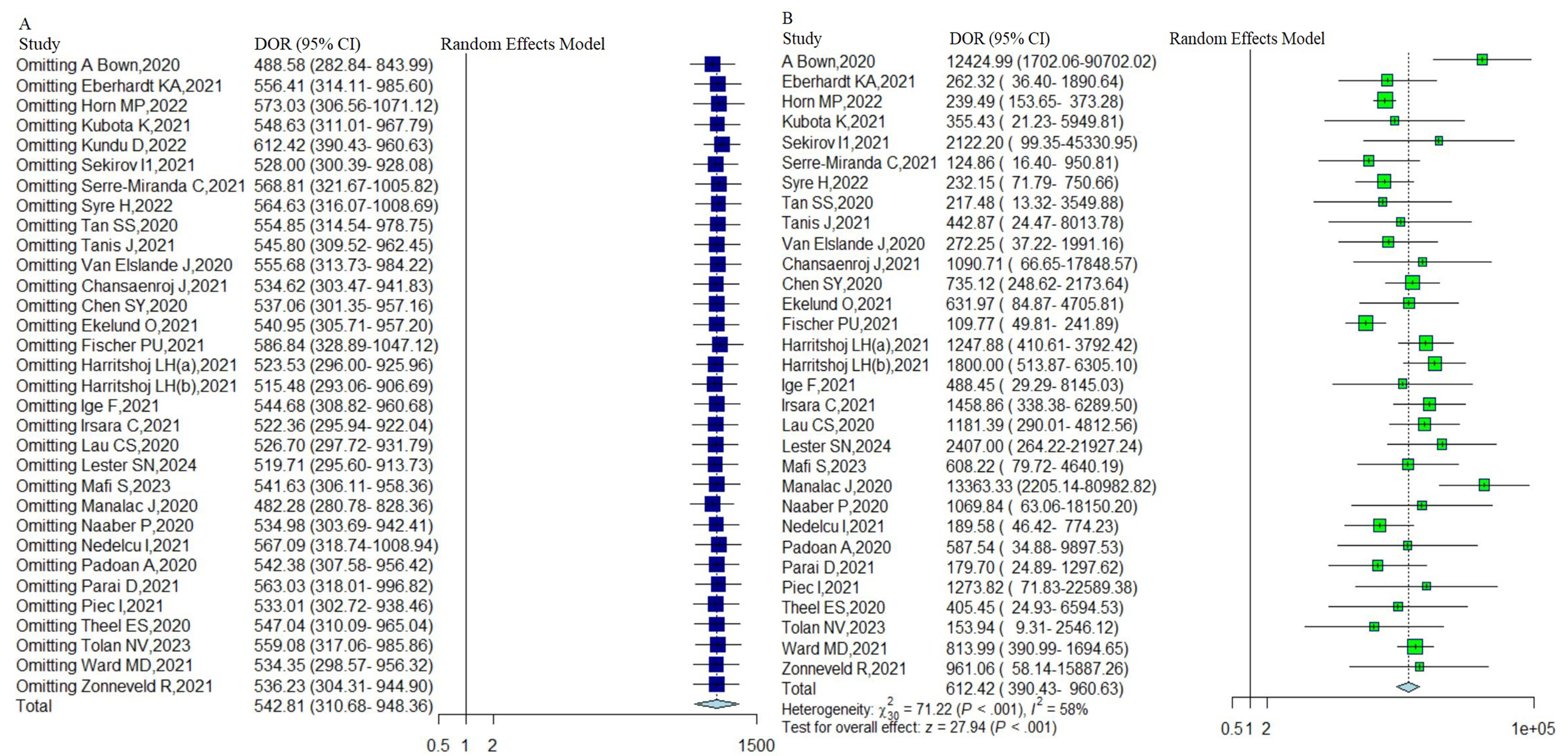


Fig. 1: Sensitivity analysis of Abbott SARS-CoV-2 IgG. Each study was omitted, and the pooled DOR of the other studies was calculated with random effects model.


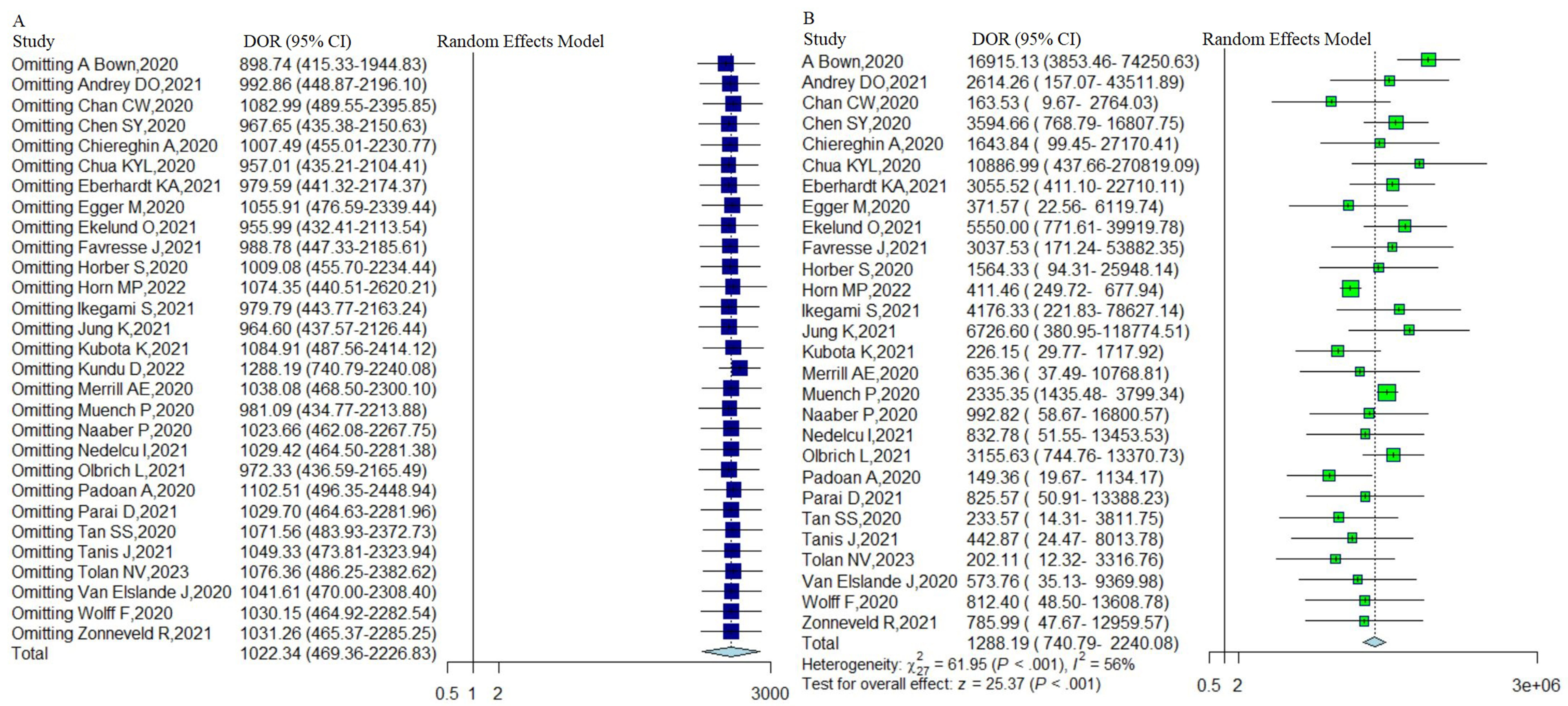


Fig. 2: Sensitivity analysis of Elecsys Anti-SARS-CoV-2 N. Each study was omitted, and the pooled DOR of the other studies was calculated with random effects model.


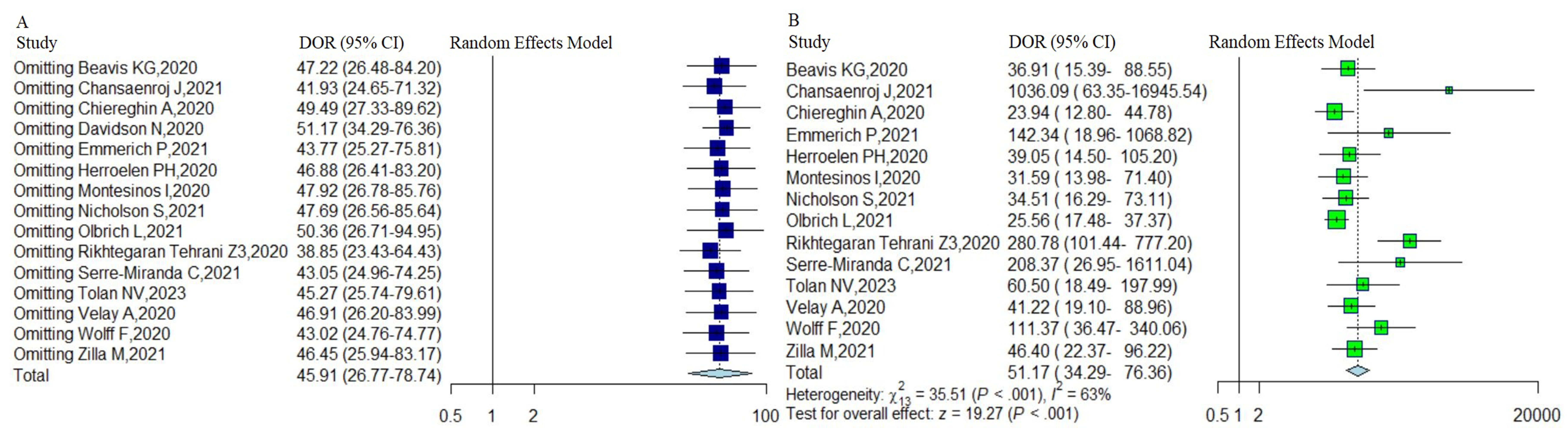


Fig. 3: Sensitivity analysis of Euroimmun Anti-SARS-CoV-2 IgA. Each study was omitted, and the pooled DOR of the other studies was calculated with random effects model.


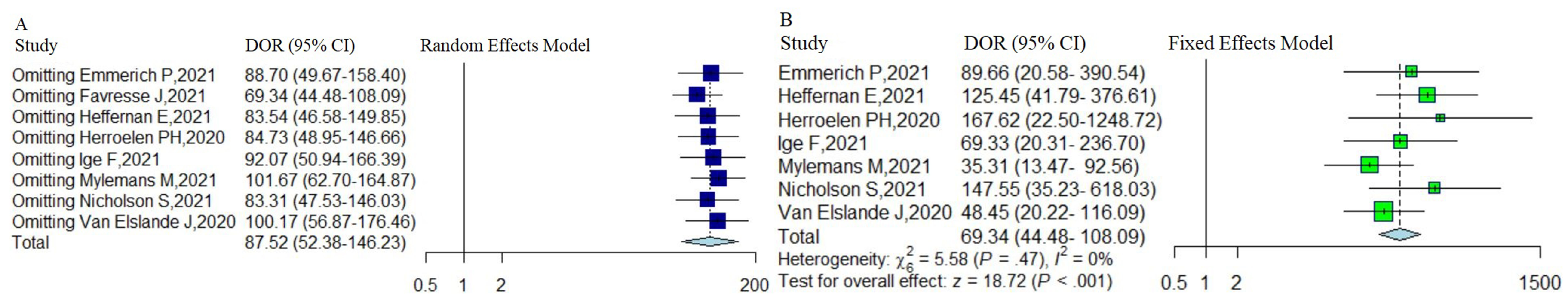


Fig. 4: Sensitivity analysis of Euroimmun Anti-SARS-CoV-2 N-IgG. Each study was omitted, and the pooled DOR of the other studies was calculated with fixed effects model.
