# Supplementary figures and images for: An Indirect Comparison of Diagnostic Accuracy for Seven Different SARS‐CoV‐2 Serological Assays: A Meta‐Analysis and Adjusted Indirect Comparison of Diagnostic Test Accuracy
Source: Influenza Other Respir Viruses. 2025 Sep 9;19(9):e70155. doi: 10.1111/irv.70155 (PMC12418076; doi:10.1111/irv.70155)

**Appendix 6.** Funnel chart of Deek’s test.


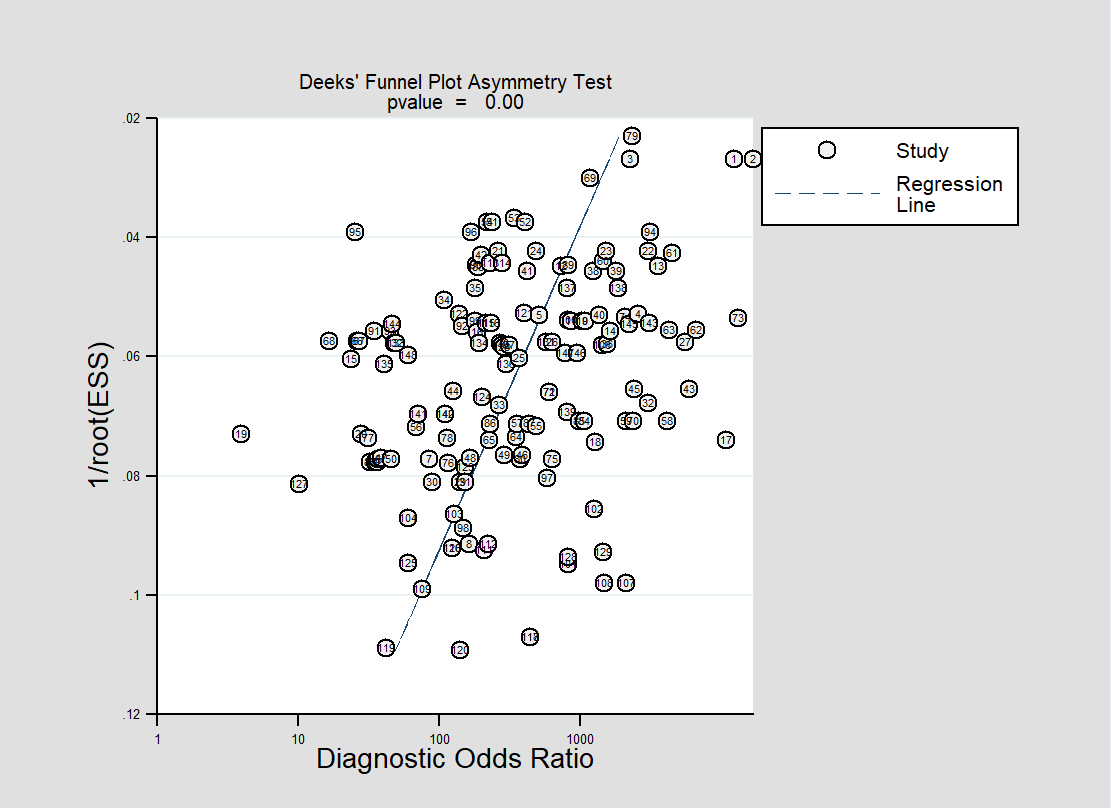

Supplement: Supplementary file 6 — Appendix S6: Funnel chart of Deek's test. (DOC) [file IRV-19-e70155-s004.doc]
